# miR21 modulates the Hippo signaling pathway via interference with PP2A Bβ to inhibit trophoblast invasion and cause preeclampsia

**DOI:** 10.1016/j.omtn.2022.09.006

**Published:** 2022-09-20

**Authors:** Mingyu Hu, Yangxi Zheng, Jiujiang Liao, Li Wen, Juan Cheng, Jiayu Huang, Biao Huang, Li Lin, Yao Long, Yue Wu, Xuan Ye, Yong Fu, Hongbo Qi, Philip N. Baker, Chao Tong

**Affiliations:** 1 State Key Laboratory of Maternal and Fetal Medicine of Chongqing Municipality, The First Affiliated Hospital of Chongqing Medical University, Chongqing 400016, China; 2 Department of Stem Cell Transplantation and Cell Therapy, MD Anderson Cancer Center, Houston, TX 77054, USA; 3 Department of Obstetrics, Chongqing Women and Children’s Hospital of Chongqing Medical University, 401147, China; 4 Reproductive Medicine Center, The First Affiliated Hospital of Chongqing Medical University, Chongqing 400016, China; 5 College of Life Sciences, University of Leicester, Leicester LE1 7RH, UK

**Keywords:** MT: Non-coding RNAs, preeclampsia, miR21, PP2A Bβ, hippo, trophoblast, invasion

## Abstract

Preeclampsia (PE) is a pregnancy-specific disorder attributed to deficient extravillous trophoblast (EVT) invasion into the uterus, but the mechanism of EVT invasion remains unclear. In this study, we found significantly elevated expression of microRNA 21 (miR21), which negatively regulates trophoblast invasion and migration, in preeclamptic placentae. Whole-genome RNA sequencing revealed that *PPP2R2B*, which encodes PP2A Bβ, and the Hippo pathway are downstream targets of miR21. The effects of miR21 on trophoblast mobility were abolished in LATS1^T1079A/S909A^ and YAP-5SA mutants. Moreover, we found that PP2A Bβ dephosphorylates LATS1 via direct protein-protein interactions and thus modulates the phosphorylation and subcellular distribution of YAP. *PPP2R2B* overexpression ameliorated the miR21-induced LATS1-YAP phosphorylation and cytoplasmic sequestration of YAP, which resulted in the rescue of compromised trophoblast invasion and migration. The upregulation of placental miR21 abundance by placenta-specific nanoparticles loaded with agomir-miR21 during placentation interfered with *PPP2R2B* and activated the Hippo pathway in the placenta, leading to a PE-like phenotype. Thus, aberrant elevation of miR21 impairs EVT mobility by modulating the PP2A Bβ/Hippo axis, which is one of the causes of PE.

## Graphical abstract

**Figure:**
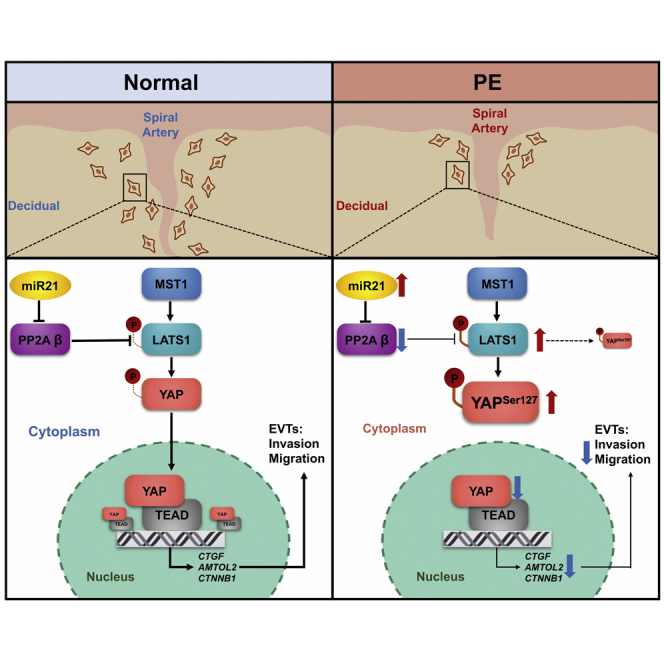

## Teaser

Our study highlights the importance of the miR21/PP2A Bβ/Hippo axis in the regulation of trophoblast invasion and migration, thus providing in-depth insights into the etiology of PE from the perspective of posttranscriptional gene regulation.

## Introduction

Preeclampsia (PE) is a leading complication of pregnancy characterized by new-onset hypertension and proteinuria at ≥20 weeks of gestation.^1^ This multisystem disorder affects up to 4%–5% of pregnancies worldwide and leads to a series of adverse perinatal outcomes^2^ that are mainly attributed to preterm delivery,^3^ which occurs secondary to maternal or fetal complications, intrauterine growth restriction (IUGR), and fetal death. PE is believed to be an ischemic placental disease^4^ that results from impaired spiral artery remodeling and inadequate trophoblast invasion.^5^^,^^6^ However, the pathophysiological mechanisms of dysfunctional migration and invasion of extravillous trophoblasts (EVTs) in PE remain to be elucidated.

MicroRNAs (miRNAs) are a subset of 20- to 24-nucleotide-long noncoding RNAs that cause degradation of targeted genes or translational inhibition at the posttranscriptional level.^7^ miRNAs are involved in numerous important biological events, including placental development,^8^ tumorigenesis,^7^ and cardiac disease.^9^ Growing evidence indicates that dysregulation of miRNAs is correlated with trophoblastic dysfunction and PE development.^10^^,^^11^ Nevertheless, the role of placental miRNAs in the pathogenesis of PE remains unclear.

Emerging studies have suggested that certain miRNAs, such as miR23a and miR199b, target protein phosphatase 2A (PP2A).^12^^,^^13^ PP2A is a ubiquitously expressed and highly conserved serine threonine phosphatase that regulates biological functions by dephosphorylating core cellular molecules in many cellular processes, such as cell proliferation, cytoskeleton dynamics, and signaling pathways.^14^ The trimeric form of PP2A is an active holoenzyme complex composed of three subunits: scaffold (A), catalytic (C), and regulatory (B) subunits. The regulatory B subunit is the predominant regulator of the PP2A holoenzyme and determines the substrate specificity and intracellular localization of the enzyme. Previous research has demonstrated that the invasion of trophoblasts into the uterus and the development of the placenta are similar to tumorigenesis to a certain extent.^15^ Moreover, cytokine receptors participate in many inflammatory diseases, including PE, and are also involved in the pathogenesis of autoimmune diseases.^16^ Although PP2A has been intensively studied in cancer^17^ and autoimmune diseases,^18^ its role in placental development or pregnancy complications such as PE needs to be further explored.

Increasing evidence has revealed that Hippo pathway proteins might be regulated by PP2A as its substrates.^17^ The mammalian Hippo pathway is a highly conserved pathway that regulates tissue homeostasis, organ size, and stem cell renewal and participates in tumor initiation or progression.^19^ The key components of the Hippo pathway kinase cascade include mammalian sterile 20-like kinase 1/2 (MST1/2), which phosphorylates and activates the downstream kinase large tumor suppressor 1/2 (LATS1/2) and the final transcriptional regulator Yes-associated protein 1 (YAP). YAP is a critical transcriptional coactivator that translocates between the cytoplasm and the nucleus; this protein can modulate target gene expression and thereby tumorigenesis and metastasis of most solid tumors. Once the cytoplasmic Hippo kinase module is active (Hippo ON), active MST1/2 (p-MST1^Thr183^/MST2^Thr180^) promotes phosphorylation of the LATS1/2 kinases (p-LATS1^Thr1079^ and p-LATS2^Thr1041^). Active LATS1/2 then phosphorylates YAP on various residues, and Ser127 (of YAP) is the predominant residue for its deactivation. In the absence of phosphorylated MST1/2 and LATS1/2, dephosphorylated YAP can translocate into the nucleus to act as a transcriptional coactivator.^19^ Furthermore, accumulating evidence has shown that LATS1 functions as a novel regulator in cellular homeostasis.^20^^,^^21^ YAP is also expressed in the human placenta, which suggests its involvement in placental development.^22^ Given that placental development shares substantial similarities with tumorigenesis,^15^ elucidation of the regulatory role of PP2A in the Hippo pathway in trophoblasts will contribute to our understanding of the etiology of PE.

In this study, we found aberrant upregulation of miR21 expression in the placenta of pregnancies complicated by PE, which resulted in suppression of PP2A Bβ and thus decreased dephosphorylation of LATS1, and these effects ultimately lead to YAP hyperphosphorylation and sequestration in the cytoplasm. These data provide novel insights into the role of the miR21-PP2A Bβ-Hippo signaling axis in the pathogenesis of PE.

## Results

### Trophoblasts from pregnancies complicated by PE show upregulated expression of miR21, which impairs invasion and migration

**Figure 1:**
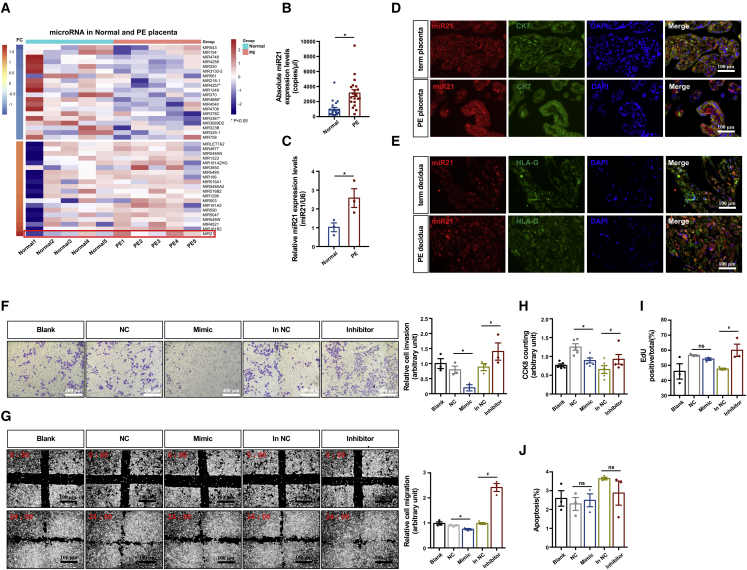
PE-complicated trophoblasts are associated with upregulated expression of miR21, which impairs invasion and migration (A) Heatmap of gene expression from a microarray of normal and preeclamptic term placentae; n = 5, two-tailed t test, ∗p < 0.05. (B) ddPCR analysis of miR21 expression in human term placentae from normal and PE-complicated pregnancies; n = 19 in the control and n = 20 in the PE group, two-tailed t test, ∗p < 0.05. (C) RT-qPCR analysis of miR21 expression in primary human trophoblasts (PHTs) isolated from normal and preeclamptic term placentae; n = 3, two-tailed t test, ∗p < 0.05. FISH of miR21 and IF staining of CK7 and HLA-G in term normal and PE-complicated placentae (D) and decidua (E); scale bar, 100 μm. HTR8/SVneo cells transfected with mimic NC (NC), inhibitor NC (in NC), miR21 mimic (mimic), or miR21 inhibitor (inhibitor) for 6 h and then cultured in fresh medium for 48 h and blank control cells were subjected to (F) Matrigel Transwell assays (scale bar, 400 μm), (G) wound-healing assays (scale bar, 100 μm), (H) CCK-8 staining assays, (I) EdU assay, and (J) flow cytometry assay for measuring apoptosis by staining with Annexin V-FITC and PI (n = 3 in each group, one-way ANOVA and Tukey’s multiple comparison test; ns, nonsignificant; ∗p < 0.05 versus mimic NC; #p < 0.05 versus inhibitor NC). The data are presented as the means ± SEMs.

To investigate the involvement of miRNAs in PE, we identified differentially expressed miRNAs between normal and matched preeclamptic placentae by microarrays (Figure 1A) and found that miR21 was the top differentially expressed miRNA (fold change = 1.82, p < 0.05). The expression levels of this miRNA were further validated in normal and preeclamptic placental samples (Table 1) by droplet digital PCR (ddPCR), which confirmed upregulated miR21 expression in the preeclamptic placentae (Figure 1B). Primary human trophoblasts (PHTs) were isolated, and increased miR21 levels were found in PHTs from the PE group (Figure 1C). An analysis combining fluorescence *in situ* hybridization (FISH) and immunofluorescence (IF) staining demonstrated that placental miR21 is expressed in various types of trophoblasts and shows upregulated expression in EVTs from PE-complicated pregnancies (Figures 1D and 1E).

**Table 1:** Clinical characteristics of the subjects

Parameters	Normal	PE	p value
Age (years)	29.4 ± 0.54	30.1 ± 0.61	0.3978
Gestational age (weeks)	35.83 ± 0.40	34.89 ± 0.26	0.0568
Systolic pressure (mm Hg)	110.5 ± 1.71	155.4 ± 3.22	<0.0001∗
Diastolic pressure (mm Hg)	75.8 ± 0.77	100.7 ± 2.58	<0.0001∗
Body mass index	24.09 ± 0.22	24.54 ± 0.38	0.3180
Fetal birth weight (g)	3291 ± 90.25	2758 ± 65.42	<0.0001∗

The data are presented as the means ± SEMs, two-tailed t test, ∗p < 0.05.

Because the immortalized human trophoblast line HTR-8/SVneo expresses a high level of miR21 (Figure S1A), we manipulated miR21 abundance in HTR-8/SVneo cells by transfection with mimic and inhibitor (Figure S1B). Matrigel-based assays and scratch assays showed that both the invasion and migration of HTR-8/SVneo cells were significantly inhibited by the miR21 mimic but stimulated by the inhibitor (Figures 1F and 1G). Compared with the remarkable inhibitory effect of miR21 on the invasion and migration of trophoblasts, its effects on cell proliferation or apoptosis appeared less significant (Figures 1H–1J).

### miR21 regulates the subcellular distribution of YAP in trophoblasts by modulating phosphorylation

**Figure 2:**
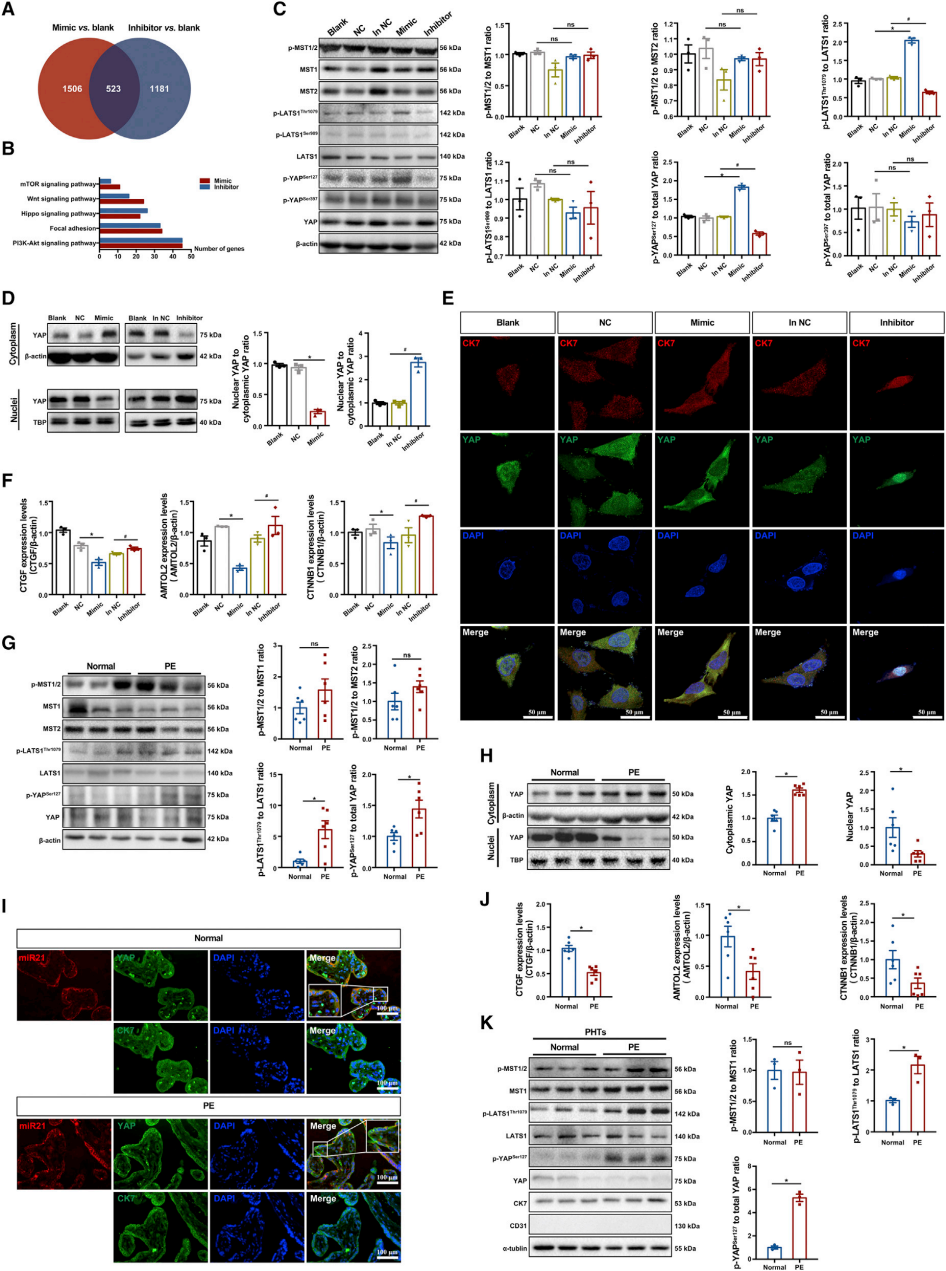
miR21 regulates the subcellular distribution of YAP in trophoblasts by modulating phosphorylation (A) Venn analysis and (B) GO enrichment analysis of biological process of mRNAs decreased by miR21 mimic and increased by miR21 inhibitor in HTR8/SVneo cells as determined by whole-genome RNA sequencing. HTR8/SVneo cells transfected with miR21 mimic or miR21 inhibitor and blank and scramble controls were subjected to (C) western blotting of p-MST1/2, MST1, MST2, p-LATS1^Thr1079^, p-LATS1^Ser909^, LATS1, p-YAP ^Ser127^, p-YAP ^Ser397^, and YAP; (D) western blotting of YAP in cytoplasm and nuclei (n = 3 in each group, one-way ANOVA and Tukey’s multiple comparison test; ns, nonsignificant; ∗p < 0.05 versus mimic NC; #p < 0.05 versus inhibitor NC); (E) IF staining of YAP (green), CK7 (red), and DAPI (blue) (scale bar, 50 μm); and (F) RT-qPCR of *CTGF*, *AMOTL2*, and *CTNNB1* (n = 3 in each group, one-way ANOVA and Tukey’s multiple comparison test; ns, nonsignificant; ∗p < 0.05 versus mimic NC; #p < 0.05 versus inhibitor NC). (G) Western blotting of p-MST1/2, MST1, MST2, p-LATS1^Thr1079^, LATS1, p-YAP ^Ser127^, and YAP in normal and preeclamptic term placentae; n = 6, two-tailed t test; ns, nonsignificant; ∗p < 0.05. (H) Western blotting of YAP in cytoplasm and nuclei of normal and PE term placentae; n = 6, two-tailed t test, ∗p < 0.05. (I) FISH of miR21 (red) and IF costaining of YAP (green) in normal (upper) and preeclamptic (lower) term placentae. Trophoblasts were stained for CK7 (green) on serial sections, and nuclei were counterstained by DAPI (blue); scale bar, 100 μm. (J) RT-qPCR of *CTGF*, *AMTOL2*, and *CTNNB1* in normal and preeclamptic term placentae; n = 6, two-tailed t test, ∗p < 0.05. (K) Western blotting of p-MST1/2, MST1, p-LATS1^Thr1079^, LATS1, p-YAP^Ser127^, and YAP in PHTs isolated from normal and preeclamptic term placentae; n = 3, two-tailed t test; ns, nonsignificant; ∗p < 0.05. The data are presented as the means ± SEMs.

To elucidate the underlying regulatory mechanism of the effects of miR21 on trophoblast function, we subjected HTR-8/SVneo cells with upregulated and downregulated abundance of miR21 to whole-genome RNA sequencing. In comparison with the wild-type (WT) group, the group with upregulated miR21 level showed changes in 3,715 mRNAs, including 1,686 that exhibited upregulated expression and 2,029 that showed downregulated expression. In contrast, interference with miR21 elevated the levels of 1,704 mRNAs and suppressed the expression of 1,957 mRNAs (Figure 2A). A Gene Ontology (GO) enrichment analysis of differentially expressed genes decreased by the miR21 mimic and enhanced by the miR21 inhibitor revealed that the phosphoinositide 3-kinase-protein kinase B (PI3K-Akt) signaling pathway, focal adhesion, and the Hippo signaling pathway were influenced by miR21 regulation (Figure 2B).

Recent work by our group and other groups has demonstrated that YAP, a key protein in the Hippo pathway, plays a critical role in the maintenance of invasive trophoblasts^23^ and is thus needed for expansion of the human placenta.^22^ Moreover, the PI3K-Akt, Wnt, and mammalian target of rapamycin (mTOR) signaling pathways have been linked to Hippo signaling,^24^^,^^25^ whereas focal adhesion has been shown to be closely correlated with trophoblast migration and invasion.^26^ Together, these findings prompted us to determine whether the regulatory effects of miR21 on trophoblast invasion and migration involve the Hippo pathway.

Interestingly, our results showed that upregulation of miR21 elevated the levels of p-LATS1^Thr1079^ and p-YAP^Ser127^, but not p-LATS1^Ser909^ and p-YAP^Ser397^, in HTR8/SVneo cells. Suppression of miR21 level specifically diminished the levels of p-LATS1^Thr1079^ and p-YAP^Ser127^ (Figure 2C). Nevertheless, changes in miR21 abundance did not significantly interfere with MST1/2 phosphorylation. Because dephosphorylated YAP can be transported to the nucleus to facilitate gene transcription, whereas phosphorylated YAP is retained in the cytoplasm,^23^ we next investigated whether the phosphorylation status of YAP in response to miR21 expression was associated with its subcellular redistribution in trophoblasts. Western blotting analysis demonstrated that cytoplasmic YAP was increased in miR21-overexpressing HTR8/SVneo cells, whereas nuclear YAP was sharply decreased (Figure 2D). Similarly, IF staining showed marked retention of cytoplasmic YAP in the presence of the miR21 mimic, whereas the miR21 inhibitor induced notable accumulation of YAP in the nuclei of HTR8/SVneo cells (Figure 2E). These results demonstrated an inverse correlation between miR21 expression and the nuclear localization of YAP in trophoblasts. Furthermore, the expression levels of downstream target genes of YAP, including *CTGF*,^23^ *AMTOL2*, and *CTNNB1*, were negatively correlated with miR21 regulation (Figure 2F).

Consistent with our findings in HTR8/SVneo cells, human preeclamptic placentae with upregulated miR21 expression exhibited significantly higher levels of p-LATS1^Thr1079^ and p-YAP^Ser127^ than the controls (Figure 2G), but the p-MST1/2 levels did not differ. Additionally, significantly elevated cytoplasmic YAP levels and decreased nuclear YAP levels were observed in the preeclamptic placentas (Figure 2H). The reduction in YAP expression observed in the nuclei of preeclamptic placental tissue was further confirmed by IF staining (Figure 2I), and the transcription of *CTGF*, *AMTOL2*, and *CTNNB1* was found to be significantly compromised in the preeclamptic placentae (Figure 2J). Moreover, the p-LATS1^Thr1079^ and p-YAP^Ser127^ levels were significantly elevated in PHTs of the preeclamptic placentae, and the phosphorylation of MST1/2 remained similar in the PE and normal groups (Figure 2K).

Altogether, the above-described evidence strongly indicated that aberrant miR21 elevation leads to activation of the Hippo pathway in trophoblasts, resulting in the sequestration of YAP in the cytoplasm and subsequent suppression of downstream genes.

### The modulation of trophoblast invasion and migration by miR21 is dependent on LATS1 or YAP phosphorylation

**Figure 3:**
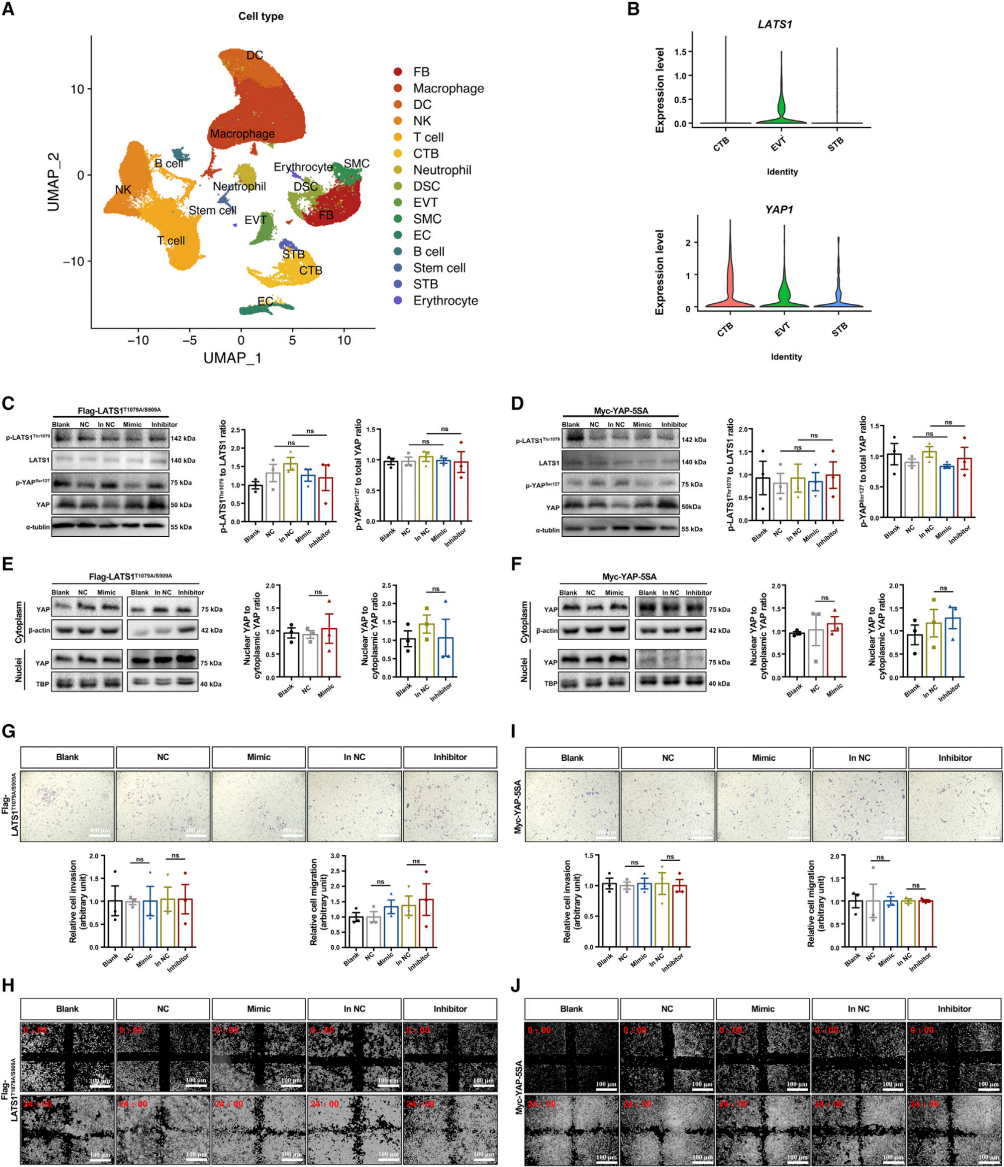
The modulation of trophoblast invasion and migration by miR21 is dependent on phosphorylation of LATS1 or YAP (A) Representative UMAP for six samples. FB, fibroblasts; DCs, dendritic cells; NK cells, natural killer cells; CTBs, cytotrophoblasts; DSCs, decidual stromal cells; EVT, extravillous trophoblast; SMCs, smooth muscle cells; EC, endothelial cell; STB, syncytiotrophoblast. (B) Violin plots for LATS1 (upper) and YAP1 (lower) in different cell types of trophoblasts. FLAG-LATS1^T1079A/S909A^ cells and Myc-YAP-5SA cells were transfected with mimic NC (NC), inhibitor NC (in NC), miR21 mimic (mimic), or miR21 inhibitor (inhibitor) for 6 h and then cultured in fresh medium for 48 h before any treatments and measurements. A blank control was included. Western blotting of p-LATS1^Thr1079^, LATS1, p-YAP^Ser127^, and YAP in (C) FLAG-LATS1^T1079A/S909A^ cells and (D) Myc-YAP-5SA cells. Western blotting of YAP in the cytoplasm and nuclei of (E) FLAG-LATS1^T1079A/S909A^ cells and (F) Myc-YAP-5SA cells. (G) Matrigel Transwell assay (scale bar, 400 μm) and (H) wound-healing assay (scale bar, 100 μm) of FLAG-LATS1^T1079A/S909A^ cells. (I) Matrigel Transwell assay (scale bar, 400 μm) and (J) wound-healing assay (scale bar, 100 μm) of Myc-YAP-5SA cells; one-way ANOVA and Tukey’s multiple comparison test; ns, nonsignificant. The data are presented as the means ± SEMs.

By 10× single-cell RNA sequencing of placentae and deciduae from three healthy subjects, we generated a transcriptomic resource of 44,790 cells and identified 15 cell clusters (Figures 3A and S2). This study is the first to reveal the different expression patterns of *LATS1* and *YAP1* in different cell types of the human placenta. The expression levels of *YAP1* and *LATS1* in EVTs were abundant (Figure 3B), indicating that the Hippo pathway may play a crucial role in EVTs. Moreover, these expression patterns of LATS1 and YAP in EVTs were then validated by IF staining of decidual cells from normal and preeclamptic pregnancies. Intriguingly, the colocalization of YAP and DAPI staining was compromised in EVTs of preeclamptic pregnancies compared with those of normal pregnancies, but this difference was not found for LATS1 (Figures S3A and S3B). Notably, the expression of miR21, LATS1, and YAP in human primary EVTs indicated their involvement in the biological regulation of trophoblasts during early placentation (Figure S3C).

To further determine whether phosphorylation of the LATS1-YAP signaling axis mediates the impact of miR21 on trophoblast invasion and migration, we generated an HTR-8/SVneo cell line that constitutively expressed an inactive (unphosphorylated) form of LATS1 (FLAG-LATS1^T1079A/S909A^), in which Thr1079 and Ser909 were mutated to alanine (Figure S4A). Moreover, we confirmed that the introduced FLAG-LATS1^T1079A/S909A^ competitively bound to YAP in HTR-8/SVneo cells (Figure S4B). In addition, we established an HTR-8/SVneo cell line with constitutively active YAP (Myc-YAP-5SA) that expresses a YAP protein bearing five serine-to-alanine mutations (S61A, S109A, S127A, S164A, S381A). These mutations are reportedly resistant to phosphorylation and cytoplasmic sequestration,^27^ even though they bind to LATS1 in HTR-8/SVneo cells (Figures S4C and S4D).

miR21 abundance in FLAG-LATS1^T1079A/S909A^ and Myc-YAP-5SA mutant HTR8/SVneo cells was then increased by the mimic or suppressed by the inhibitor (Figures S4E and S4F). Neither overexpression nor inhibition of miR21 altered the phosphorylation of LATS1^Thr1079^, LATS1^Ser909^, or YAP^Ser127^ in these cells (Figures 3C, 3D, S5A, and S5B). Accordingly, both the accumulation of cytoplasmic YAP induced by miR21 overexpression and the retention of nuclear YAP induced by miR21 inhibition were blunted in the FLAG-LATS1^T1079A/S909A^ and Myc-YAP-5SA cells (Figures 3E and 3F); this effect was confirmed by IF staining (Figures S5C and S5D). These results demonstrated that phosphorylation of LATS1^Thr1079^ and YAP ^Ser127^ is needed for the miR21-induced redistribution of YAP in trophoblasts.

To further confirm the involvement of LATS1 and YAP phosphorylation in the miR21-mediated regulation of trophoblastic function, we treated FLAG-LATS1^T1079A/S909A^ and Myc-YAP-5SA cells with a miR21 mimic or inhibitor and then performed cell invasion and migration assays. The modulation of miR21 level disturbed neither invasiveness nor migration (Figures 3G–3J) and did not significantly interfere with the proliferation and apoptosis of the FLAG-LATS1^T1079A/S909A^ and Myc-YAP-5SA cells (Figures S5E–S5J). These results indicated that the miR21-induced phosphorylation of LATS1 and YAP is essential for the inhibitory regulation of trophoblast invasion and migration.

### The regulation of trophoblast invasion by miR21 is dependent on the dephosphorylation of LATS1^Thr1079^ and YAP^Ser127^ via suppression of PP2A Bβ, which interacts with LATS1

The Hippo signaling pathway maintains regulatory function by balancing the phosphorylation and dephosphorylation of its components between kinases and phosphatases.^28^ Several phosphatases, such as PP2A, protein phosphatase 1 (PP1), striatin-interacting phosphatases and kinases (STRIPAK), and protein tyrosine phosphatase nonreceptor type 14 (PTPN14), reportedly interact with the Hippo pathway to regulate cell proliferation and migration.^17^

**Figure 4:**
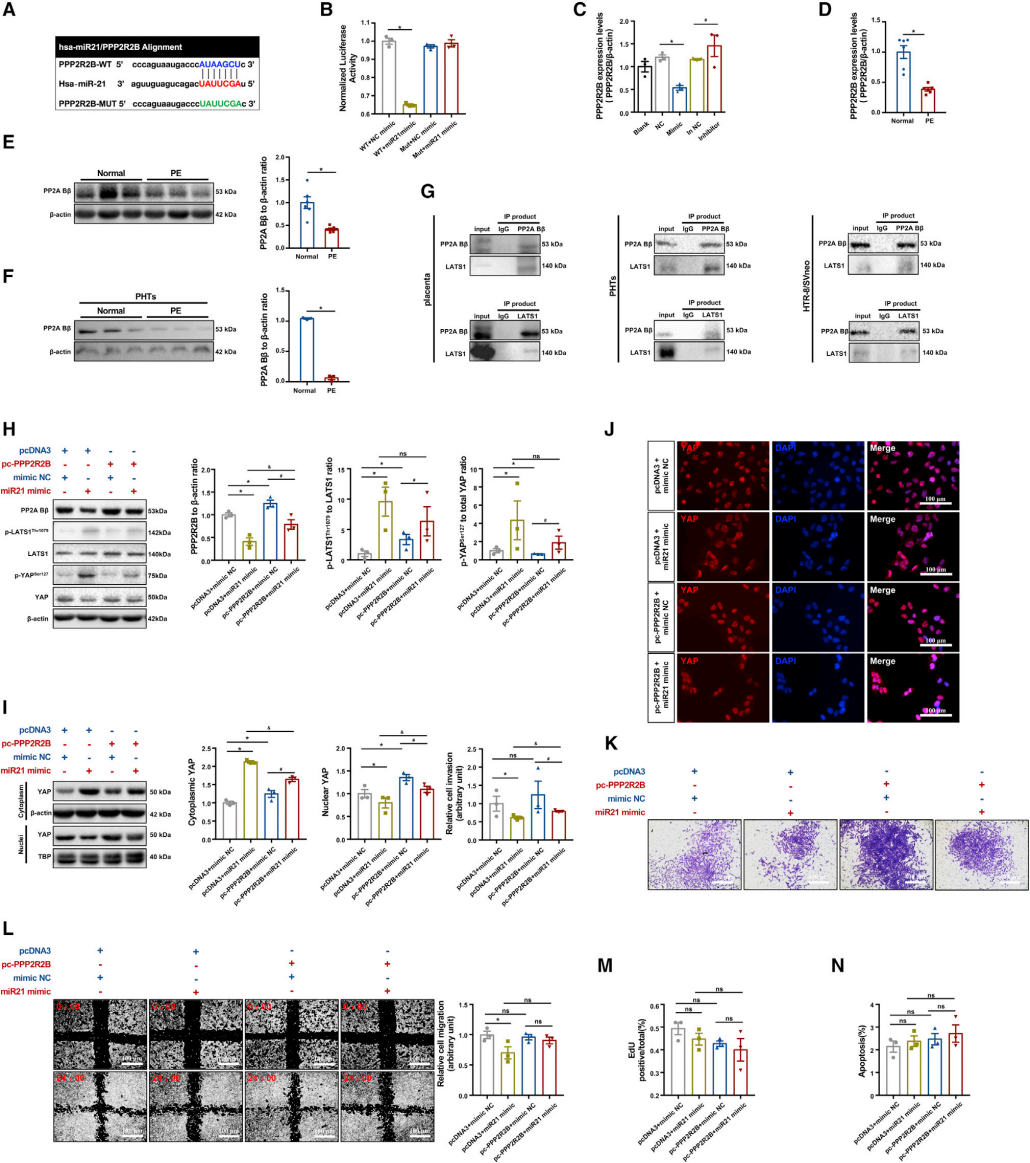
The regulation of trophoblast invasion by miR21 is dependent on dephosphorylation at LATS1^Thr1079^ and YAP^Ser127^ through the suppression of PP2A Bβ, which interacts with LATS (A) Schematic representation of the putative miR21-binding sites in *PPP2R2B*. (B) Luciferase reporter assay of HTR8/SVneo cells cotransfected with *PPP2R2B* WT or MUT reporter plasmids with miR21 mimic or mimic NC; n = 3, one-way ANOVA and Tukey’s multiple comparison test, ∗p < 0.05 versus the *PPP2R2B* WT + mimic NC group (WT + NC mimic). (C) RT-qPCR of *PPP2R2B* in HTR8/SVneo cells transfected with miR21 mimic or miR21 inhibitor; n = 3, one-way ANOVA and Tukey’s multiple comparison test, ∗p < 0.05 versus mimic NC; #p < 0.05 versus inhibitor NC. (D) RT-qPCR of *PPP2R2B* in normal and preeclamptic term placentae; n = 6 in each group, two-tailed t test, ∗p < 0.05. (E) Western blotting of PP2A Bβ in normal and preeclamptic term placentae; n = 6, two-tailed t test, ∗p < 0.05. (F) Western blotting of PP2A Bβ in PHTs; n = 3, two-tailed t test, ∗p < 0.05. Reciprocal coIP of (G) PP2A Bβ and LATS1 in human placenta (left), PP2A Bβ and LATS1 in PHTs (middle), and PP2A Bβ and LATS1 in HTR8/SVneo cells (right). pcDNA3 or pc-PPP2R2B plasmid-transfected HTR8/SVneo cells were cotransfected with mimic NC or miR21 mimic for 6 h, cultured in fresh medium for 48 h, and then subjected to (H) western blotting of PP2A Bβ, p-LATS1^Thr1079^, LATS1, p-YAP^Ser127^, and YAP. (I) Western blotting of YAP in cytoplasm and nuclei. (J) IF staining of YAP (red) and DAPI (blue) (scale bar, 100 μm); (K) Matrigel Transwell assay (scale bar, 400 μm); (L) wound-healing assay (scale bar, 100 μm); (M) EdU assay; and (N) flow cytometry for the measurement of apoptosis by staining with Annexin V-FITC and PI; n = 3, one-way ANOVA and Tukey’s multiple comparison test; ns, nonsignificant; ∗p < 0.05 versus the pcDNA3 + mimic NC group; #p < 0.05 versus the pc-PPP2R2B + mimic NC group; and p < 0.05 versus the pcDNA3 + miR21 mimic group. The data are presented as the means ± SEMs.

Pertinently, the involvement of the protein serine/threonine phosphatase (PSP) family in the regulation of the Hippo pathway has been well documented.^29^, ^30^, ^31^ Our RNA sequencing of HTR8/SVneo cells with miR21 upregulation and silencing revealed a differentially expressed mRNA, *PPP2R2B*, which encodes PP2A Bβ, the regulatory subunit of PP2A. We then conducted luciferase-based reporter assays to validate the putative binding between miR21 and *PPP2R2B*. Moreover, the mutation was introduced in the putative target sequence to prevent miR21 interaction (Figure 4A).

Our data showed that the miR21 mimic significantly reduced the luciferase activity of the WT rather than mutant (MUT) PPP2R2B plasmids (Figure 4B), confirming the direct binding of miR21 with *PPP2R2B*. To validate the putative negative regulatory effect of miR21 on *PPP2R2B* expression, we measured the *PPP2R2B* expression levels and found that the mRNA levels in HTR8/SVneo cells were downregulated by the miR21 mimic but upregulated by the inhibitor (Figure 4C). Accordingly, the *PPP2R2B* mRNA levels were significantly lower in preeclamptic placentas than in placentas of uncomplicated pregnancies (Figure 4D).

We then measured the PP2A Bβ protein levels in whole placenta lysate and PHTs from PE-complicated pregnancies and found that the PP2A Bβ protein levels in these pregnancies were significantly lower than those in uncomplicated pregnancies (Figures 4E and 4F). PP2A modulates the Hippo pathway by dephosphorylating its component proteins,^30^^,^^32^ thus, to ascertain whether PP2A Bβ is directly involved in the regulation of the Hippo pathway by miR21, we verified the interaction between PP2A Bβ and Hippo pathway molecules. Coimmunoprecipitation (coIP) using human placental tissues, PHTs, and HTR8/SVneo cells showed that PP2A Bβ physically binds with LATS1 (Figure 4G) but not MST1 or YAP (Figures S6A–S6F), which indicates that miR21 may regulate the Hippo pathway in trophoblasts via the PP2A Bβ/LATS1 axis.

To verify the putative role of PP2A Bβ in mediating the miR21-induced activation of the Hippo pathway in trophoblasts, we established *PPP2R2B*-overexpressing HTR8/SVneo cells via transfection with pc-PPP2R2B plasmids. These cells exhibited a 2-fold increase in the PP2A Bβ protein levels (Figure S7A). Nevertheless, PP2A Bβ overexpression alone had no significant impact on the viability of trophoblasts but markedly promoted invasion (Figures S7B–S7F). Cotransfection of the miR21 mimic with the pc-PPP2R2B plasmids showed that the decreased dephosphorylation of LATS1^Thr1079^ and YAP^Ser127^ in HTR8/SVneo cells via the miR21-mediated downregulation of PP2A Bβ was notably diminished by overexpression of PP2A Bβ (Figure 4H). Moreover, PP2A Bβ overexpression led to significant accumulation of nuclear YAP, while concurrently alleviating cytoplasmic YAP retention caused by upregulation of miR21 (Figures 4I and 4J). Consistent with our observed changes in Hippo signaling, the inhibition of cell invasion and migration due to miR21 was significantly rescued by upregulated PP2A Bβ expression (Figures 4K and 4L), and this rescue did not affect cell viability (Figures 4M and 4N). These results suggest that miR21 regulates trophoblast invasion and migration by suppressing PP2A Bβ.

### The administration of an agomir-miR21 nanoparticle specifically elevates placental miR21 expression in mice

**Figure 5:**
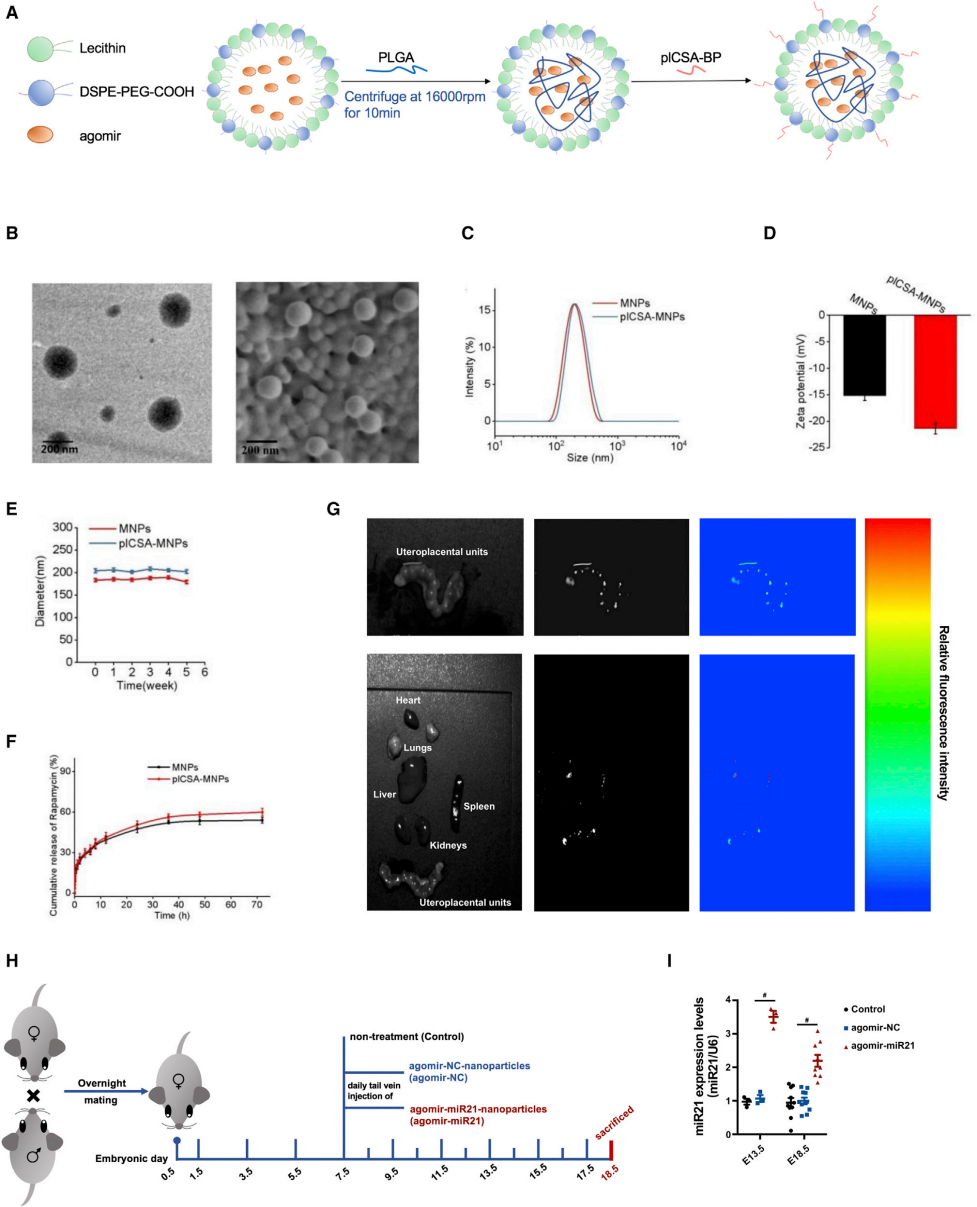
The administration of agomir-miR21 nanoparticles specifically elevated the abundance of miR21 in the mouse placenta (A) Schematic illustration of nanoparticle synthesis. (B) Representative transmission electron microscopy (TEM) and scanning electron microscopy images of nanoparticles; scale bar, 200 nm. (C) Size distribution profiles of nanoparticles. (D) Electronegative property of nanoparticles. (E) Stability of nanoparticles in serum. (F) *In vitro* release profiles of nanoparticles in PBS (pH 7.4) at 37°C. (G) Pregnant mice were injected with agomir-miR21-Cy3-nanoparticles (800 μmol/kg) via the tail vein on E9.5 and imaged. (H) Schematic illustration of the experimental design. (I) RT-qPCR of miR21 in placentae collected on E13.5 and E18.5 from dams in the control, agomir-NC, or agomir-miR21 groups; n = 3 in each group on E13.5; n = 10 in the control and agomir-NC groups on E18.5, n = 9 in the agomir-miR21 group on E18.5, two-way ANOVA and Tukey’s multiple comparison test. The data are presented as the means ± SEMs.

To validate the regulatory effects of miR21 on PP2A Bβ/Hippo and cell invasion *in vivo*, we developed placental chondroitin sulfate A (CSA)-binding peptide (plCSA-BP)-conjugated nanoparticles loaded with methotrexate (plCSA-MNPs) according to previously described methods^33^ for specific delivery to the mouse placenta (Figure 5A). Transmission electron microscopy (TEM) and scanning electron microscopy revealed that the nanoparticles displayed spherical morphologies (Figure 5B) with an approximate mean diameter of 190 nm (Figure 5C). Moreover, these nanoparticles showed electronegative properties with a zeta potential distribution of plCSA-MNPs of nearly −21.4 ± 1.027 mV (Figure 5D). An assessment of stabilities *in vitro* demonstrated that these nanoparticles exhibited a relatively narrow change in size in 10% fetal bovine serum (FBS) over nearly 5 weeks, indicating strong dispersal and long-term stability (Figure 5E). Moreover, the *in vitro* release profiles of the nanoparticles showed a rapid release of cargo within the first 24 h and a sustained release from 24 to 70 h, indicating efficient release capabilities (Figure 5F).

Next, to verify the delivery specificity of these nanoparticles loaded with agomir-miR21 to the placenta, we loaded placenta-specific nanoparticles with agomir-miR21-Cy3 and administered them to pregnant mice on embryonic day 9.5 (E9.5) via intravenous injection. We detected signals from the agomir-miR21-Cy3 nanoparticles in the placenta 1 h after injection by *ex vivo* fluorescence imaging (Figure 5G).

Based on the 60% loading capacity of our nanoparticles, nanoparticles containing gradient doses of agomir-miR21 (100, 200, 400, or 800 μmol/kg) were then suspended in 300 μL of phosphate-buffered saline (PBS) and administered to pregnant mice via tail vein injection daily during E7.5–E9.5 (i.e., in early placentation). The specificity of agomir-miR21 delivery to the placenta was further confirmed by measuring the abundance of miR21 in the major organs of the dams and fetuses. miR21 abundance was only upregulated in the placenta and exhibited a dose-dependent increase (Figures S8A–S8J). Then, 800 μmol/kg agomir-miR21 nanoparticles or an equivalent amount of agomir-NC nanoparticles were administered to pregnant mice daily during E7.5–E9.5 (experimental design illustrated in Figure 5H). Our data showed that treatment with agomir-miR21 nanoparticles rather than agomir-NC nanoparticles resulted in significantly higher miR21 levels in mouse placentae collected on both E13.5 and E18.5 (Figure 5I), indicating that the administration of agomir-miR21 nanoparticles during early pregnancy could effectively upregulate placental miR21 level until late pregnancy.

### The increase in placental miR21 abundance during early pregnancy induces a PE-like phenotype in mice

**Figure 6:**
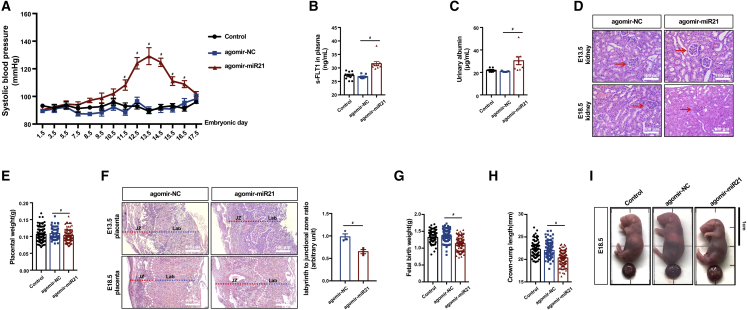
The increase in placental miR21 abundance during early pregnancy induces a PE-like phenotype in mice (A) Systolic blood pressure of pregnant mice in the agomir-NC, agomir-miR21, and control groups; n = 10, two-way ANOVA and Tukey’s multiple comparison test. (B) Plasma s-FLT1 of pregnant mice in the control, agomir-NC, and agomir-miR21 groups determined by ELISA; n = 10 in each group, one-way ANOVA and Tukey’s multiple comparison test. (C) Urinary albumin assay of pregnant mice; n = 7 in the control and agomir-NC groups, n = 8 in the agomir-miR21 group, one-way ANOVA and Tukey’s multiple comparison test. (D) H&E staining of maternal kidneys collected on E13.5 and E18.5; scale bar, 100 μm. (E) Placental weight; n = 106 placentae from 10 dams in the control group, n = 97 placentae from 10 dams in the agomir-NC group, and n = 111 placentae from 10 dams in the agomir-miR21 group, one-way ANOVA and Tukey’s multiple comparison test. (F) H&E staining of the placental morphology in the uteroplacental units collected at E13.5 and E18.5 from the agomir-NC and agomir-miR21 groups; n = 3, two-tailed t test, ∗p < 0.05. (G) Fetal weight; n = 106 fetuses from 10 dams in the control group, n = 97 fetuses from 10 dams in the agomir-NC group, and n = 111 fetuses from 10 dams in the agomir-miR21 group, one-way ANOVA and Tukey’s multiple comparison test. (H) Crown-rump length (CRL) of fetuses; n = 97–111, one-way ANOVA and Tukey’s multiple comparison test. (I) Representative images of the fetuses of the control, agomir-NC, and agomir-miR21 groups; scale bars, 1 cm. The data are presented as the means ± SEMs.

**Figure 7:**
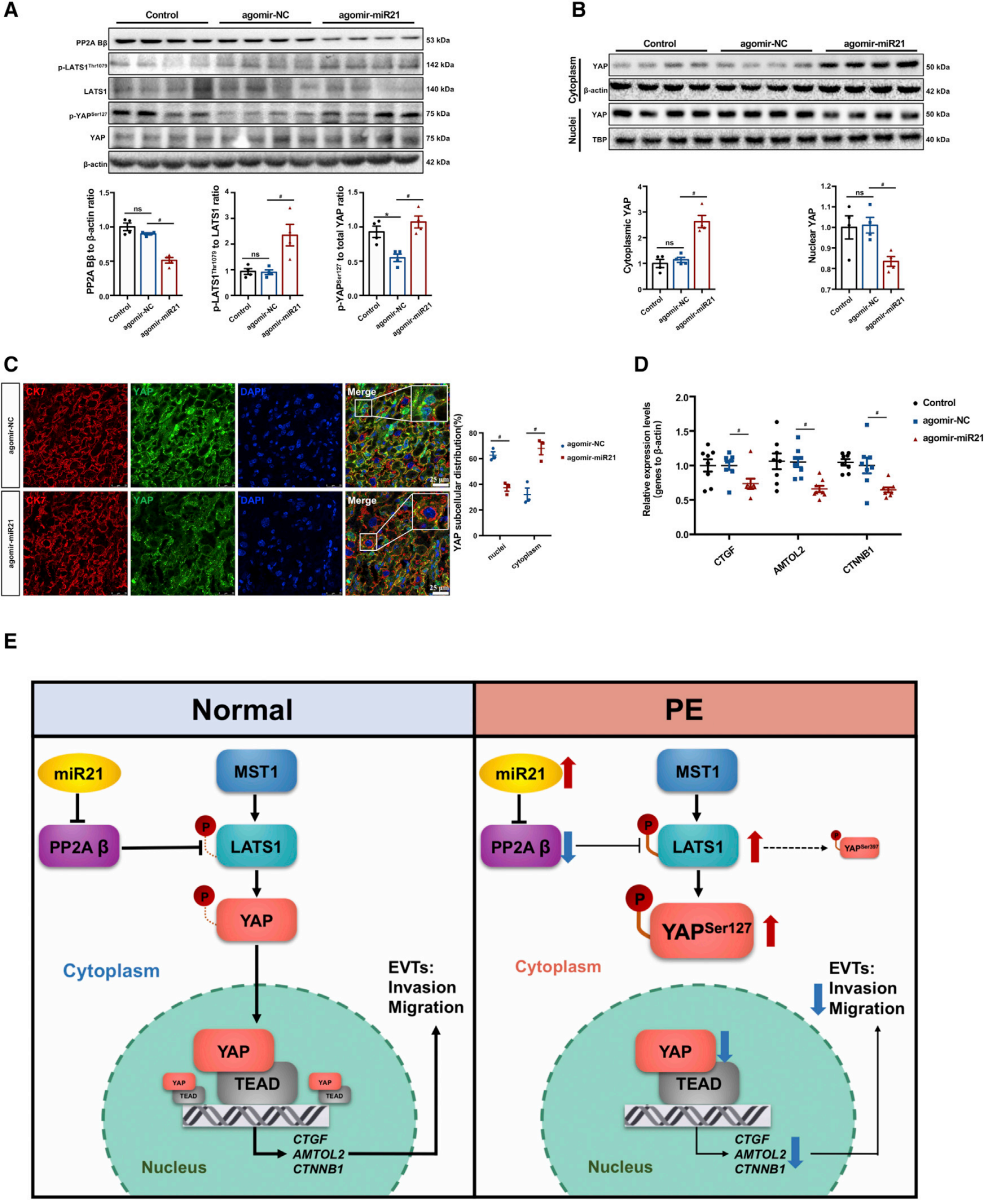
The upregulation of miR21 reduced the dephosphorylation of LATS1^Thr1079^ and YAP^Ser127^ by suppressing PP2A Bβ *in vivo* Western blotting of (A) PP2A Bβ, p-LATS1 (Thr1079), LATS1, p-YAP (Ser127), and YAP in placentae at E18.5 and (B) cytoplasmic and nuclear YAP in placentae at E18.5 (n = 4 placentae from four dams in the agomir-NC, agomir-miR21, and control groups; one-way ANOVA and Tukey’s multiple comparison test; ns, nonsignificant). (C) IF staining of CK7(red) and YAP (green) in placentae of the agomir-NC (upper) or agomir-miR21 group (lower) at E18.5. Nuclei were counterstained by DAPI (blue); scale bar, 25 μm; two-way ANOVA and Tukey’s multiple comparison test, ∗p < 0.05. (D) RT-qPCR of *ctgf*, *amotl2*, and *ctnnb1* in mouse placentae at E18.5, n = 6 placentae from six dams in each group, two-way ANOVA and Tukey’s multiple comparison test; ns, nonsignificant; ∗p < 0.05 versus the control group; #p < 0.05 versus the agomir-NC group. (E) Schematic representation of the mechanism underlying the miR21/PP2A Bβ/Hippo axis in extravillous trophoblast (EVT) invasion and placental development. The data are presented as the means ± SEMs.

According to a previously described mouse model, the placenta-specific delivery of agomir-miR21 could substantially elevate the systolic blood pressure of pregnant mice (Figure 6A). However, this elevation was not observed in nonpregnant mice (Figure S9). Moreover, agomir-miR21 nanoparticle administration led to a significant increase in soluble FLT1 (sFLT1) (Figure 6A), a well-known antiangiogenic factor that has been implicated in the pathogenesis of PE.^34^ Pertinently, augmented urinary albumin levels were detected in the agomir-miR21 group (Figure 6C). Notably, a reduction in the glomerulus open capillary area was observed on E18.5 but not on E13.5 (Figure 6D), indicating that the PE-like phenotype observed in the agomir-miR21 nanoparticle treatment group was not nephrogenic but rather placental in origin. In addition, the agomir-miR21 group demonstrated a lower placental weight (Figure 6E). H&E staining of the placentae collected at E13.5 and E18.5 revealed that the ratio of the labyrinth area (Lab) to the junctional zone (JZ) was decreased in the agomir-miR21 group due to a reduction of the Lab and vacuolization of the JZ (Figure 6F). This decrease indicated that upregulation of miR21 led to aberrant development of the placentae. Furthermore, agomir-miR21 treatment led to fetal growth restriction, as manifested by a significantly lower birthweight and crown-rump length (CRL) (Figures 6G–6I). Together, these data indicated that specifically upregulating placental miR21 abundance during placentation induces a PE-like syndrome. Consistent with our findings from *in vitro* models and human placenta, specific upregulation of miR21 in mouse placenta led to a significant reduction in the PP2A Bβ levels and decreased the dephosphorylation of LATS1 and YAP (Figure 7A). Moreover, upregulated miR21 led to YAP retention in the cytoplasm, which resulted in the attenuation of nuclear localization (Figures 7B and 7C) and downregulation of *CTGF*, *AMTOL2*, and *CTNNB1* expression (Figure 7D).

## Discussion

Accumulating evidence suggests that dysregulation of miRNAs in the placenta is involved in the pathogenesis of PE.^10^ Nevertheless, the expression of miR21 in the placentae of pregnancies complicated by PE remains controversial: some studies have found an association between preeclamptic placentae and downregulated miR21 expression,^35^ and other studies have reported opposing findings.^36^^,^^37^ In the present study, the upregulation of miR21 expression in preeclamptic human placentae was first identified via unbiased high-throughput microarray screening and then confirmed by ddPCR, which measures the absolute number of transcript copies.

Given the recognized interaction between miRNAs and long noncoding RNAs (lncRNAs), previous studies have suggested potential explanations for the correlation between abnormal levels of miR21 in PE and lncRNAs. lncRNA taurine-upregulated gene 1 (TUG1), as a miR21 sponge,^38^ promotes migration and invasion via miR-29b in trophoblasts.^39^ Moreover, lncRNA maternally expressed gene 3 (MEG3), another lncRNA reportedly implicated in miR21 modulation,^38^ has been shown to serve as a positive regulator in trophoblasts and is suppressed by miR210 in PE.^40^ However, the involvement of TUG1, MEG3, or other lncRNAs in the pathogenesis of PE through miR21 requires further investigation.

The mechanism of miR21 in the downstream regulatory network that drives the development of PE requires further study. Since the development of PE has long been attributed to loss of trophoblast invasion and consequent spiral artery remodeling deficiencies,^4^ we speculate that excessive miR21 in the placenta may contribute to this process. First, we revealed that placental miR21 is predominantly expressed in various trophoblasts. We then found that miR21 indeed inhibits trophoblast invasion and migration *in vitro* and may thus contribute to the development of PE. A previous study demonstrated that the Forkhead box M1 (FOXM1) mRNA and protein levels are decreased in preeclamptic placentas, whereas the expression of miR21 is upregulated. The results confirmed that miR21 might alter trophoblast proliferation by affecting FOXM1, which might participate in promoting the development of PE.^41^ Although we observed that upregulation of miR21 moderately inhibited trophoblast proliferation and promoted apoptosis, consistent with this report, the marked inhibition of trophoblast invasion and migration by miR21 cannot be fully explained through its relatively subtle impact on cell viability.

To determine the functions of miR21 *in vivo*, we generated a placenta-specific miR21 overexpression mouse model using a novel nanotechnology-based drug delivery system. Compared with traditional strategies for generating tissue-specific transgenic mice or systemic administration of the miR21 mimic, our approach demonstrated unparalleled advantages in cost and time savings. In addition, our model shows markedly higher bioavailability and specificity compared with systemic administration of the miR21 mimic. Most importantly, this mouse model confirmed that the specific upregulation of miR21 in placentae during placentation impairs placental development and induces PE-like features.

Because excessive miR21 can inhibit trophoblast invasion *in vitro* and induce a PE-like phenotype *in vivo*, further investigation of the molecular mechanisms regulated by miR21 is needed. miRNAs play critical roles in posttranscriptional gene regulation by destabilizing mRNAs containing complementary base sequences; therefore, aberrant expression of a single miRNA may result in marked changes in the transcriptome.^42^ To elucidate the molecular basis underlying the regulatory mechanisms of miR21 in trophoblast motility, we profiled transcriptome changes in miR21-overexpressing and miR21-underexpressing HTR8/SVneo cells by whole-genome RNA sequencing. The results suggested that *PPP2R2B*, which encodes PP2A Bβ, a subunit of PP2A phosphatases, is a putative downstream target of miR21.

Phosphatases have previously been reported to participate in various biological processes. Functional PP2A is a trimer consisting of regulatory subunits (B) that bind with catalytic (C) and scaffolding subunits (A), which participate in tumor progression. The B regulatory subunit determines the substrate specificity of the holoenzyme.^17^ Several studies have demonstrated that the transcriptional regulation of PP2A by miRNAs usually involves targeting of different regulatory subunits.^43^ Although PP2A is generally recognized as a tumor suppressor with genetic alterations or functional inactivation in cancer, previous studies have revealed that the PP2A B subunit promotes cell proliferation in various cancer cells.^32^^,^^44^ Here, we demonstrated that upregulation of the expression of PP2A Bβ alone did not significantly affect cell viability but promoted trophoblast invasion, consistent with the results from previous research in tumorigenesis.^45^

According to our whole-genome RNA sequencing results, the Hippo pathway may be a critical signaling pathway involved in the regulation of PP2A Bβ in trophoblasts. The phosphorylation cascade of kinases in the Hippo pathway is partially regulated by PP2A.^46^ Thus, we hypothesized that the interference of *PPP2R2B* by miR21 may influence the dephosphorylation-mediated regulation of PP2A on Hippo molecules and ultimately alter Hippo pathway activity. Key components of the Hippo pathway, such as phosphorylation of kinase MST1 and LATS1, result in phosphorylation-dependent cytoplasmic retention of the transcriptional coactivator YAP by the 14-3-3 proteins. This phosphorylation MST/LATS/YAP cascade is reportedly involved in tumorigenesis and tightly regulated by phosphatases, including PP2A.^17^

Tang et al. reported that striatin3 (STRN3), encoded by *PPP2R6B*, directly interacts with MST2 in HEK293FT cells.^47^ These researchers further revealed a distinct binding site for STRN3 on PP2A subunit A, specifically a short C-terminal portion of the coiled coil of STRN3 (composed of amino acid residues termed STRN3Core).^48^ Moreover, a recent study demonstrated that the inhibition of catalytic subunit C reduced vessel growth by inactivating YAP in endothelial cells^49^ and thus provide new insights into the involvement of PP2A and the Hippo pathway in placental development. Here, we report that PP2A Bβ interacts with LATS1 rather than MST1 or YAP in human placental tissues, PHTs, and HTR8/SVneo cells, which has been proposed in cancer research.^32^^,^^48^ This finding identified a novel regulatory mechanism of YAP activation that relies on PP2A Bβ. However, the molecular basis and features underlying the interaction between PP2A Bβ and LATS1 warrant further study.

Moreover, we found that the phosphorylation of LATS1 at Thr1079 rather than Ser909 responds to miR21 abundance. The phosphorylation of LATS1 at Thr1079 led to the cytoplasmic retention of YAP *in vitro* and *in vivo*. Moreover, we demonstrated that YAP phosphorylation at Ser127 rather than Ser397 occurs in response to miR21 in trophoblasts; p-YAP^Ser127^ is involved in YAP cytoplasmic retention, whereas p-YAP^Ser397^ is correlated with proteasomal degradation.^32^ Considering this information, we focused on p-YAP^Ser127^ in miR21-regulated YAP localization. As expected, upregulated miR21 levels in preeclamptic placentae, mouse placentae or HTR8/SVneo cells suppressed PP2A Bβ and enhanced p-YAP^Ser127^. Subsequently, the expression levels of downstream genes of YAP, such as *CTGF*, *AMTOL2*, and *CTBNN1*, were downregulated due to reductions in nuclear YAP.

In summary, abnormal elevation of miR21 during placentation interferes with PP2A Bβ, which leads to decreased dephosphorylation of LATS1 and YAP. Compromised inhibition, in turn, impedes the cytoplasmic-to-nuclear translocation of YAP and subsequent gene transcription involved in trophoblast invasion and migration, which ultimately causes PE (Figure 7E). Our findings highlight the importance of the miR21-mediated degradation of *PPP2R2B* mRNA leading to decreased PP2A Bβ phosphatase abundance in the regulation of trophoblast invasion and migration and thus provide in-depth insights into the etiology of PE from the perspective of posttranscriptional gene regulation.

## Materials and methods

### Ethics statement

This study involving patients and animals was approved by the Ethics Committee of the First Affiliated Hospital of Chongqing Medical University (no. 2018-108) in accordance with the principles set out in the Declaration of Helsinki. All samples were collected with written informed consent provided by the participants. The animal procedures were conducted in accordance with the Guidelines of Chongqing Medical University and approved by the Ethics Committee of the First Affiliated Hospital of Chongqing Medical University.

### Patient and sample collection

Placental and decidual tissues were collected from women with PE (n = 20) with normotensive pregnancies admitted to the First Affiliated Hospital of Chongqing Medical University for elective cesarean deliveries. PE was diagnosed according to the guidelines of the American College of Obstetrics and Gynecology (ACOG).^50^ Patients with other major pregnancy complications, such as infection, gestational diabetes mellitus (GDM), chronic hypertension, immune diseases, other gestational complications, and chronic health conditions, were excluded. Individuals with noninfective premature deliveries were recruited to match the gestational age of PE. The clinical characteristics of the subjects are shown in Table 1. Placental villous tissue (n = 10) was collected from women who underwent legal termination for nonmedical reasons during the first trimester (6–9 weeks), and patients with a history of spontaneous abortion or ectopic pregnancy were excluded. Placental specimens were randomly collected as previously described by Yang et al.^51^ immediately after delivery and then washed with cold PBS, flash frozen in liquid nitrogen and stored at −80°C for further use, or fixed in 4% formaldehyde.

### Animal

Eight- to 12-week-old CD-1 female mice weighing 25–35 g from the Experimental Animal Center of Chongqing Medical University were mated with age-matched male mice. Upon observation of a vaginal plug, the day of mating was considered E0.5. All mice were kept in a temperature-controlled room (23°C) with a 12-h light:12-h dark cycle.

### Intravenous injection via the caudal vein

Pregnant mice were randomly assigned to three groups (control, n = 12; agomir-NC, n = 12; agomir-miR21, n = 12). On E7.5, nanoparticles suspended in 300 μL of PBS were administered to pregnant mice daily through the tail vein over three consecutive days at a dose of agomir-NC or agomir-miR21 equivalent to 800 μmol/kg. The mice belonging to the control group did not receive any treatment. The mice were sacrificed on E18.5 for sample collection. All animal experiments were carried out in accordance with the National Institutes of Health guidelines for the use and care of animals and approved by the Institutional Animal Care and Use Committee of Chongqing Medical University.

### Measurement of the blood pressure

The blood pressure was measured by tail-cuff plethysmography (Visitech Systems, USA) every 2 days during E1.5–7.5 and then every day during E7.5–17.5. The mice were maintained conscious and in restraints, and 10–20 actual measurements were obtained after normalization.

### Measurement of urinary albumin

Spot urine of pregnant mice was collected at E18.5 and centrifuged at 4,000 × *g* and 4°C for 10 min, and the supernatant was collected and frozen at −80°C. Urinary albumin was measured using a Mouse Albumin ELISA Quantitation kit (Assaypro, USA) according to the manufacturer’s protocols, and the absorbances were read using a microplate reader (Thermo Fisher Scientific, USA).

### Measurement of s-FLT1

After sacrifice on E18.5, mouse plasma was collected using EDTA as an anticoagulant and centrifuged at 1,000 × *g* for 30 min. Plasma was then removed and stored in aliquots at −80°C. Plasma s-FLT1 was measured with a Mouse s-FLT1 ELISA Quantitation kit (Cloud-Clone Corp., China) according to the manufacturer’s protocols.

### H&E staining of placental and renal tissue

Placentae and kidneys were fixed in 4% paraformaldehyde, embedded in paraffin, and cut into 3-μm-thick sections. The sections were deparaffinized, rehydrated, and then stained with hematoxylin for 5 min and then with eosin for 2 min. Images were captured with an EVOS microscope (Life Technologies, USA).

### Primary trophoblast isolation

#### First trimester

PHTs were isolated from first-trimester placental villi as previously described.^52^ Briefly, immediately after legal termination, placental villus tissue (6–9 weeks of gestation, n = 3–5 per isolation) was cut into small pieces (1–3 mm). The first digestion was performed with 0.125% trypsin (Gibco, USA) for 15 min at 37°C. After the digestion was stopped with 10% FBS (Gibco, USA), the cells were filtered through a 700-μm cell strainer (Miltenyi, Germany), and the remaining tissues were digested with 0.25% trypsin for 15 min at 37°C. After two consecutive digestion steps followed by Percoll (Bio-Rad, USA) density gradient centrifugation at 300 × *g* and 4°C for 20 min, trophoblast cells were isolated. The PHTs were then seeded on 4-μg/mL fibronectin-coated dishes and fixed for IF after 24 h.

#### Third trimester

PHTs from term placental tissues were isolated as previously described.^53^ Briefly, immediately after delivery, placental tissue was rinsed in ice-cold saline and minced into small pieces (1–3 mm). For digestion of the placental tissue, 625 mg of dispase II (Roche, Switzerland) and 400 μL of DNase (Roche, Switzerland) were added and incubated for 1 h and 15 min, respectively, at 37°C. After filtering (70 μm, Miltenyi, Germany) and centrifuging at 300 × *g* and 4°C for 7 min, the precipitate was resuspended in 40 mL of platelet lysis solution (Gibco, USA) and washed twice gently with DMEM/F12 (Gibco, USA), which contained 10% FBS (Gibco, USA). The suspension was then added to a Percoll gradient (60%, 50%, 40%, 30%, and 20%, Bio-Rad, USA) and centrifuged at 1,000 × *g* for 20 min. The 20%–40% Percoll layer was collected, suspended in DMEM/F12 mixed with 10% FBS, and then centrifuged at 300 × *g* for 7 min. The pellet was resuspended in DMEM/F12 containing 10% FBS and antibiotics and then seeded onto dishes for 3 h to adhere.

### Cell culture

The immortalized human trophoblast cell line HTR8/SVneo was purchased from the American Type Culture Collection (ATCC, USA). The human choriocarcinoma cell lines JAR, JEG3, and BeWo were obtained from the Cell Bank of the Chinese Academy of Sciences. Both HTR-8/SVneo and JAR cells were cultured in Roswell Park Memorial Institute (RPMI) 1640 (Gibco, USA) containing 10% FBS (Gibco, USA) and 1% penicillin–streptomycin (Beyotime, China). JEG3 cells were cultured with DMEM/F12 medium (Gibco, USA). BeWo cells were cultured with DMEM/F12K medium (Gibco, USA). All the cells were cultured at 37°C in 5% CO_2_ humidified air.

### FISH

Cy3-hsa-miR21 (5′-TCAACATCAGTCTGATAAGCTA-3′) probes were synthesized and obtained from GenePharma (China). Hybridization assays were performed using a FISH Detection Kit (Gene Pharma, China) according to the manufacturer’s instructions. All images were captured with a fluorescence microscope (Life Technologies, USA).

### Western blotting

The detailed immunoblotting procedure was completed according to our previous study.^51^ Primary antibodies against PP2A Bβ (1:1,000, 13123-1-AP), CK7 (1:1,000, 17513-1-AP), human leukocyte antigen G (HLA-G) (1:1,000, 66447-1-Ig), anti-ɑ-tubulin (1:1,000, 11224-1-AP), and anti-β-actin (1:1,000, 66009-1-Ig) were purchased from Proteintech (China). Anti-MST1 (1:500, bs-28134R) and anti-MST2 (1:500, bs-4663R) were purchased from Bioss (China). Anti-LATS1 (1:1,000, #3477), anti-p-LATS1 Ser909 (1:1,000, #9157S), anti-p-LATS1 Thr1079 (1:1,000, #8654S), anti-YAP (1:1,000, #14074S), anti-p-YAP Ser127 (1:1,000, #13008S), and anti-p-MST1/2 (1:1,000, #49332S) were purchased from Cell Signaling Technology (USA). Anti-p-YAP Ser127 (1:5,000, ab226760) and anti-CD31 (1:1,000, ab9498) were purchased from Abcam (UK). Anti-YAP (1:500, sc-376830) was purchased from Santa Cruz (USA).

### CoIP

Anti-MST1 (Cell Signaling Technology, USA), anti-LATS1 (Cell Signaling Technology, USA), anti-YAP (Cell Signaling Technology, USA), anti-YAP (Santa Cruz, USA), anti-PP2A Bβ (Proteintech, China), anti-Myc (Proteintech, China), anti-FLAG (Proteintech, China), or anti-IgG (Santa Cruz, USA) antibodies were incubated with Protein A/G Magnetic Beads (Bimake, USA) for 4 h at 4°C. Samples were lysed with Thermo Scientific Pierce IP Lysis Buffer (Thermo Fisher Scientific, USA) with protease inhibitor mixture (Bimake, USA) and incubated with an antibody-bead complex overnight at 4°C. The immunoprecipitation products were then precipitated by the antibody-bead complex using a magnetic rack and analyzed by western blotting.

### IF staining

Placental tissues were fixed in 4% paraformaldehyde and subsequently embedded in paraffin. Serial sections (3 μm) of paraffin-embedded tissues were analyzed by IF as described elsewhere.^23^ Briefly, tissue slides were deparaffinized in xylene, rehydrated in a serial ethanol gradient, and blocked with 3% H_2_O_2_ for 10 min. The slides were then immersed in TE buffer (10 mM Tris and 1.0 mM EDTA, pH 9.0), warmed in a microwave oven at 92°C–98°C for 15 min for antigen retrieval, and cooled to room temperature. The slides were then blocked with 10% goat serum (Boster, China) for 1 h at room temperature, incubated with primary antibodies overnight at 4°C, and then incubated with fluorescence-labeled secondary antibodies (Bioservice, China) at 37°C for 1 h. The nuclei were subsequently counterstained with 4′,6-diamidino-2-phenylindole (DAPI, Boster, China) and mounted with antifade mounting medium (Boster, China). The cells were fixed with 4% paraformaldehyde, permeabilized in 0.2% Triton X-100, and blocked with 10% goat serum (Boster, China). After overnight incubation with primary antibodies at 4°C, the cells were incubated with fluorescence-labeled secondary antibodies (Bioservice, China) at 37°C for 1 h. The nuclei were stained with DAPI, and images were captured with an EVOS microscope (Life Technologies, USA) and/or confocal microscope (Zeiss, Germany).

### RNA extraction, qRT-PCR, and ddPCR

Total RNA from tissues or cells was extracted using TRIzol reagent (Invitrogen, USA) according to the manufacturer’s instructions.

For miR21 measurement, 20 ng of total RNA was first reverse transcribed using the TaqMan microRNA reverse transcription kit (Thermo Fisher Scientific, USA), and miR21 (000397, Thermo Fisher Scientific, USA) and U6 (001973, Thermo Fisher Scientific, USA) snRNA-specific primers and probes and then quantified using a TaqMan PCR kit (Thermo Fisher Scientific, USA) with a Bio-Rad CFX Manager System (Bio-Rad, USA). The expression levels relative to that of U6 were determined using the ΔΔCq method.

For mRNA quantification, 1 μg of total RNA was used for reverse transcription with a Prime Script RT reagent kit (Roche, Switzerland). Real-time PCR was then performed using SYBR Green dye (Roche, Switzerland) with an Applied Biosystems PCR cycler (Bio-Rad, USA). The primers were designed and synthesized by TaKaRa (China); β-actin was used as an internal control. The primer sequences are shown in Table 2. The reactions were incubated in a 96-well plate at 95°C for 10 min and then subjected to 40 cycles of 95°C for 10 s, 63.3°C for 30 s, and 72°C for 10 s. All experiments were performed in triplicate. The threshold cycle (Ct) value was defined as the fractional cycle number at which the fluorescence passed the fixed threshold.

**Table 2:** Primers for RT-qPCR

Genes	Sequences (5' →3′)
hsa-CTGF/CCN2	forward GGAATCGGAATCCTGTCGATTAG
	reverse GTGAGGCTACCACATTTCCTAC
hsa-AMOTL2	forward CCGCATTCCACTGGGTATAA reverse GATTAGAACACAGCCCTACCTC
hsa-CTNNB1 hsa-β-actin mmu-ctgf/ccn2 mmu-amotl2 mmu-ctnnb1 mmu-β-actin	forward TGTGAATCCCAAGTACCAGTGT reverse CGTCAGACAAAGGAGAAACATT forward TGGCACCCAGCACAATGAA reverse CTAAGTCATAGTCCGCCTAGAAGCA forward CTACCGACTGGAAGACACATTT reverse GTCCCTTACTTCCTGGCTTTAC forward GACACCCTCTCTGGACTCTAT reverse GAGGAAAGCCAACCAGTATCA forward AGCTGGCCTGGTTTGATACT reverse CCATTCCCACCCTACCAAGT forward CCACCATGTACCCAGGCATT reverse CAGCTCCAGTAACAGTCCGCC

For ddPCR, the copy numbers of miR21 were measured with ddPCR Supermix for Probes (Bio-Rad, USA) according to the manufacturer’s protocols.

### Extraction of nuclear and cytoplasmic proteins

The isolation of nuclear and cytoplasmic proteins from tissues or cells was carried out using a commercial kit (Invent, USA) according to the manufacturer’s instructions. Briefly, for nuclear isolation, 200 μL of cytoplasmic extraction buffer was added to cell dishes and incubated on ice for 5 min. The lysed cells were then scraped with a pipette tip and transferred to a prechilled 1.5-mL microcentrifuge tube. After vigorous vortexing for 15 s, the tube was centrifuged in a microcentrifuge at 16,000 × *g* and 4°C for 5 min. The supernatant (cytoplasmic lysate) was collected as the cytoplasmic protein. Subsequently, appropriate amounts of nuclear extraction buffer were added to the pellet, vortexed vigorously for 15 s, and then incubated on ice for 1 min. The process of vortexing for 15 s and incubation for 1 min was repeated four times. The nuclear extract was transferred to a prechilled filter cartridge with a collection tube and centrifuged in a microcentrifuge at 16,000 × *g* for 30 s. The cytoplasmic and nuclear lysates were stored at −80°C for further use.

### Transfection

The oligonucleotide sequences of the miR21 mimic, inhibitor, or negative control (Table 3) were purchased from GenePharma (China). pQCXIH-Myc-YAP-5SA was a generous gift from Kunliang Guan (Addgene plasmid # 33093; http://www.addgene.org/33093/. RRID: Addgene_33093). The FLAG-LATS1^T1079A/S909A^ plasmid, *PPP2R2B* reporter plasmid, and *PPP2R2B* overexpression plasmid were synthesized by Hanbio Biotechnology (China). HTR8/SVneo cells at 70% confluency were transfected with 100 nM oligonucleotides or 1 μg of plasmids in the presence of Lipofectamine 2000 (Thermo Fisher Scientific, USA) in six-well plates according to the manufacturer’s instructions.

**Table 3:** Sequences of mimic and inhibitor

Oligo	Forward 5′→ 3′	Reverse 5′ → 3′
hsa-miR21mimic	UAGCUUAUCAGACUGAUGUUGA	AACAUCAGUCUGAUAAGCUAUU
hsa-miR21 inhibitor	UCAACAUCAGUCUGAUAAGCUA	
Negative control	UUCUCCGAACGUGUCACGUTT	ACGUGACACGUUCGGAGAATT
inhibitor NC	CAGUACUUUUGUGUAGUACAA	
mmu-agomir-NC	UUCUCCGAACGUGUCACGUTT	ACGUGACACGUUCGGAGAATT
mmu-agomir-miR21	UAGCUUAUCAGACUGAUGUUG	AAACAUCAGUCUGAUAAGCUAU

### Cell Counting Kit-8 assay

HTR8/SVneo cells were seeded on 96-well plates at 5000 cells/well and transfected with each of the oligonucleotides or plasmids (miR21 mimic, inhibitor, negative control, Myc-YAP 5SA and FLAG-LATS1^T1079A/S909A^) after adhesion. The supernatant was discarded after treatment for 48 h. Base medium with 10% Cell Counting Kit-8 (CCK-8) assay buffer (MedChemExpress, USA) was then added to the plates at 100 μL/well, and, after 4 h of incubation, the samples were measured with a microplate reader (Thermo Fisher Scientific, USA) at 450 nm.

### DNA synthesis assay

The 5-ethynyl-2′-deoxyuridine (EdU) assay was performed using the Click-iTR EdU Kit (RiboBio, China) according to the manufacturer’s instructions. Specifically, cells were plated in 96-well plates and treated after adhesion. A total of 100 μL of culture medium containing 50 mM EdU was added to each well, and, 4 h later, the cells were fixed with 4% formaldehyde for 30 min. After washing, the cells were incubated with a solution in the kit for 30 min and stained with Hoechst (RiboBio, China) to identify nuclei, and images were captured with a fluorescence microscope (Thermo Fisher Scientific, USA). EdU-positive cells were determined using ImageJ 1.50i software (https://imagej.en.softonic.com/).

### Apoptosis assay

Cell apoptosis was analyzed by flow cytometry using an Annexin V-FITC kit (Beyotime, China) according to the manufacturer’s protocols. The cells were plated on six-well plates at 4 × 10^5^ cells/mL per well, harvested, and washed with PBS. The cells were then mixed with Annexin V-FITC and phosphatidylinositol propidium iodide (PI)-binding buffer for 20 min, and the mixture was then analyzed using a flow cytometer (BD Biosciences, USA).

### Matrigel invasion assay

HTR8/SVneo cells (50,000 cells/well) were resuspended in RPMI-1640 medium without FBS and seeded into the upper compartment of the invasion chamber (8 μm, BD Falcon, USA), which was coated with previously diluted Matrigel (Corning, USA) in a 24-well plate. After 24 h, the upper chambers were fixed with 4% paraformaldehyde, washed with PBS, and stained with crystal violet boric acid. The cleaned upper chambers were photographed with an EVOS microscope (Life Technologies, USA). Cell counts were calculated using ImageJ 1.50i software.

### Cell migration assay

HTR8/SVneo cells were seeded on six-well plates and grown to more than 90% confluence. A cross shape was scratched into plates with a 200-μL pipette tip, and pictures were taken at 0 and 24 h. The area of wound healing was quantified using ImageJ 1.50i software.

### Luciferase reporter assay

To generate luciferase reporter plasmids for PPP2R2B, the 3′ UTR of PPP2R2B (407 nt) containing putative miR21-binding sites was cloned into a pSI-CHECK2 vector (Sangon Biotech, China). The putative miR21 target sequences shown in panel A in Figure 4 were longer sequences, which included the putative miR21 target and a portion of the sequences preceding and following the target. A day before transfection, HTR8/SVneo cells were seeded into 12-well plates. Cells were cotransfected with miR21 mimic and the WT or MUT luciferase vectors using Lipofectamine 2000 Reagent (Thermo Fisher, USA). Forty-eight hours after transfection, the luciferase activity was measured by the Dual-Luciferase Reporter System (Promega, USA) using a fluorescence microplate reader (Thermo Fisher, USA) according to the manufacturer’s instructions.

### Transcriptome microarray

Total RNA was extracted using TRIzol reagent (Invitrogen, USA) according to the manufacturer’s instructions and then purified with Agencourt AMPure magnetic beads (Beckman Coulter, USA). Target preparation for microarray processing was performed according to the instructions provided in the GeneChip® WT PLUS Reagent Kit (Thermo Fisher Scientific, USA). After hybridization with Affymetrix Human Gene 1.0ST Array chips, the microarrays were washed, stained with streptavidin-phycoerythrin on Affymetrix Fluidics Station 450 (Affymetrix, USA), and then scanned using an Affymetrix® GeneChip Command Console installed in a GeneChip® Scanner 3000 7G (Affymetrix, USA). The microarray data were analyzed by the robust multichip analysis (RMA) algorithm using the default analysis settings and global scaling as the normalization method with Partek® Genomics Suite 6.6. The log2-transformed values of the RMA signal intensities were calculated, and differential expression analysis was further performed by one-way analysis of variance (ANOVA).

### Whole-genome RNA sequencing

HTR8/SVneo cells were grown to 60%–70% confluency and then transfected with miR21 mimic and inhibitor oligonucleotides for 48 h. Total RNA was extracted using TRIzol reagent (Invitrogen, USA), and the concentration was measured by Nanodrop 2000 UV spectroscopy (Thermo Fisher, USA). A total of 5 μg of RNA per sample was used as the input material for the transcriptome libraries. The sequencing libraries were generated with a NEBNext® Ultra™^TM^ Directional RNA Library Prep Kit for Illumina® (NEB) following the manufacturer’s recommendations. The differential expression analysis of two samples was performed using the DEGseq (2010) R package. The p value was adjusted using the q value. Significantly differential expression was defined based on q value <0.01 and |log2(fold change)|>2 as the default thresholds. A GO enrichment analysis of the target gene candidates of differentially expressed miRNAs (hereafter referred to as target gene candidates) was then performed.

### Synthesis of nanoparticles

Lecithin and 1,2-distearoyl-sn-glycero-3-phosphoethanolamine-N-maleimide (polyethylene glycol 2000) carboxylic acid (DSPE-PEG-COOH) were purchased from Avanti Polar Lipids (USA). Poly(lactide-co-glycolide) (PLGA), 1-ethyl-3-(3- dimethylaminopropyl) carbodiimide hydrochloride (EDC), N-hydroxysuccinimide (NHS), and methotrexate (MTX) were obtained from Sigma-Aldrich (USA).

First, lecithin (50 mg) contained ethanol, and DSPE-PEG-COOH (6 mg) was dissolved in deionized water. The mixture was stirred for 0.5 h at 65°C. Then, PLGA (25 mg), guanidinylated hyperbranched poly(ethyleneimine) (GPEI) (12.5 mg), and RNA (1 nm) were added, and the mixture was stirred for 2 h at room temperature and centrifuged at 16,000 rpm for 10 min; these steps were repeated three times. The resulting mixture was precooled at −80°C and then subjected to deposition and lyophilization in a vacuum dryer (EYELA, China) at −53°C.

The peptides were conjugated to DSPE-PEG-COOH using EDC and NHS. First, 18 mg of the lipid-polymer nanoparticles (NPs) were suspended in 6 mL of 0.1 M 2-morpholinothanesulfonic acid (MES) buffer (pH 5.5). For preactivation of the carboxylic group, 15 mg of EDC and 5 mg of NHS were added, and the mixture was stirred for 15 min at room temperature. Three milligrams of plCSA-BP was then dissolved in 500 μL of deionized water, and the solution was added to the reaction mixture separately. Next, 350 μL of 20× PBS was added to buffer the reaction, and the pH was maintained at 7.0–8.0. The reaction mixture was stirred overnight at room temperature. Excess peptides and other impurities, such as EDC and NHS, were removed by centrifugation at 16,000 rpm to obtain the final plCSA-conjugated nanoparticles loaded with RNA. The same procedures were used to prepare plCSA-BP-conjugated nanoparticles loaded with agomir-miR21-Cy3 or MTX. After deposition and lyophilization, the nanoparticles were stored at −20°C for further experiments and resuspended in PBS before use.

### Characterization of nanoparticles

The morphology and size of the nanoparticles were observed by TEM (Hitachi, Japan) at an acceleration voltage of 80 kV. Scanning electron microscopy images were obtained with a focused ion beam (FIB) scanning electron microscope (Zeiss, Germany). Zeta potential and size measurements were performed at 25°C using a Malvern Zetasizer Nano ZS instrument (Malvern, USA). The stability of the nanoparticles in serum was evaluated by examining the size changes of the particles in 10% FBS. The release profiles of the nanoparticles were assessed using a dialysis experiment to measure the release of MTX in PBS (pH 7.4) release medium. The dialysate was removed at different scheduled time points to measure the concentration of MTX by high-performance liquid chromatography (Agilent, USA) at 307 nm.

### Isolation of single cells from term decidua and placenta

Decidual and placental tissues were minced into approximately 0.2–1-mm^3^ cubes with scissors and digested with 10 mL of 10 mg/mL collagenase IV (Sigma, USA) solution in RPMI 1640 medium (Gibco, USA) with 10% FBS (Gibco, USA) at 37°C for 90 min in a shaking incubator. The supernatant was diluted with medium and filtered through 100-μm, 70-μm, and 40-μm cell strainers (Miltenyi, Germany) in sequence. The flow-through was centrifuged and resuspended in 5 mL of red blood cell lysis buffer (Biosharp, China) for 8 min. The mixture was centrifuged at 300 × *g* and 4°C for 5 min, and the cell pellet was resuspended in 1 mL of medium for 10× single-cell sequencing.

### 10× Single-cell RNA sequencing data analysis

A Cell Ranger Single-Cell Software Suite (version 3.0, 10x Genomics) was used to align and quantify 10× sequencing data. Alignment, filtering, barcode counting, and unique molecular identifier (UMI) counting were performed with a Cell Ranger count module to generate a feature-barcode matrix and determine clusters.

Seurat (version 4.0.1) was used to analyze downstream data. Cells with a gene number lower than 500 or higher than 8,000 or with a mitochondrial gene ratio higher than 30% were regarded as abnormal and filtered out. All Seurat objects for individual samples were integrated into one combined object. The union of the top 2,000 variable genes for combined objects was then used to perform canonical correlation analysis (CCA) of different samples of data. The CCA subspaces were then aligned using 1:25 CCA dimensions, and uniform manifold approximation and projection (UMAP) was performed to visualize all the cells. The expression of established lineage marker genes was used to assign cell types. The gene expression levels were visualized using VlnPlot.

### Statistics

All data were collected using Prism 7 software (GraphPad). The data in bar and line graphs represent the means ± standard errors of the mean (SEMs). Two-tailed Student’s t test was used for comparisons between two groups. For comparisons among multiple groups, one-way ANOVA followed by Tukey’s multiple comparisons test was applied. For multiple groups with multiple characteristics, two-way ANOVA was used. All the data are presented as the means ± SEMs, and a p value <0.05 was considered to indicate statistical significance.

## Data Availability

The data and materials described in the manuscript will be available upon reasonable request made to the corresponding authors (delivery charges and agreement of usage may apply).

## Supplemental information

Document S1. Figures S1–S 9

Document S2. Article plus supplemental information
