# Supplementary material for: miR21 modulates the Hippo signaling pathway via interference with PP2A Bβ to inhibit trophoblast invasion and cause preeclampsia
Source: Mol Ther Nucleic Acids. 2022 Sep 20;30:143–61. doi: 10.1016/j.omtn.2022.09.006 (PMC9547189; doi:10.1016/j.omtn.2022.09.006)
Supplement: Document S1. Figures S1–S — 9 [file mmc1.pdf]

OMTN, Volume *30*

Supplemental information

miR21 modulates the Hippo signaling pathway via interference with PP2A Bβ to inhibit trophoblast invasion and cause preeclampsia

Mingyu Hu, Yangxi Zheng, Jiujiang Liao, Li Wen, Juan Cheng, Jiayu Huang, Biao Huang, Li Lin, Yao Long, Yue Wu, Xuan Ye, Yong Fu, Hongbo Qi, Philip N. Baker, and Chao Tong

Supplemental figures

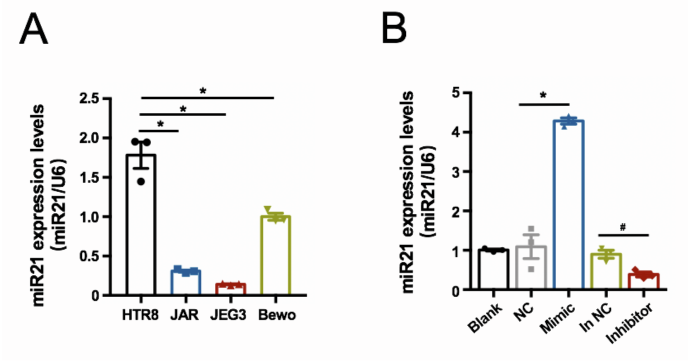

Figure S1. Examination of miR21 abundance in trophoblast cell lines.

(A) RT-qPCR of miR21 in various trophoblast cell lines, n=3, one-way ANOVA and Tukey's multiple comparison test, *p<0.05 vs. HTR8/SVneo; (B) HTR8/SVneo cells transfected with mimic NC (NC), inhibitor NC (in NC), miR21 mimic (mimic) or miR21 inhibitor (inhibitor) for 6 h, followed by culturing in fresh media for 48 h. Transfected cells with a blank control were subjected to RT-qPCR analysis of miR21 expression; n=3 in each group, one-way ANOVA and Tukey's multiple comparison test, *p<0.05 vs. mimic NC; #p<0.05 vs. inhibitor NC; data are presented as the mean ± SEM.

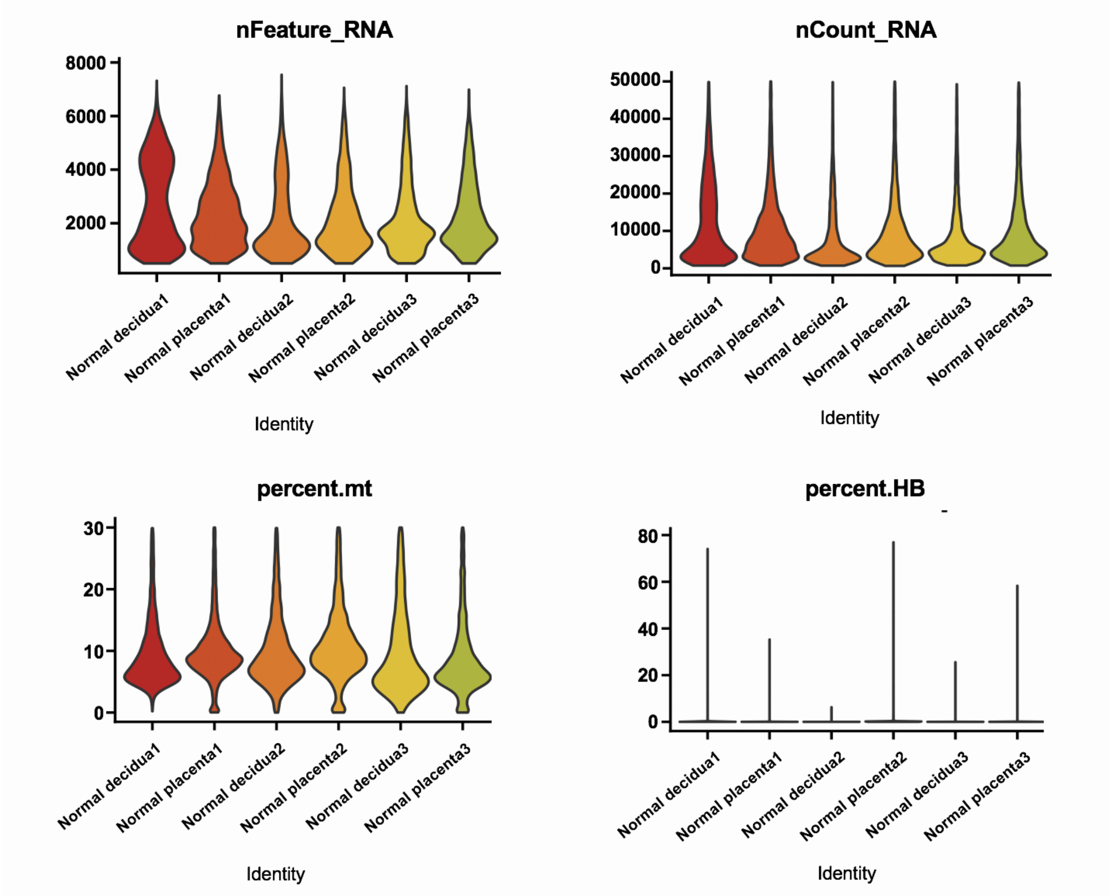

Figure S2. Quantity control (QC) of 10× single-cell sequencing in placenta and decidua from healthy controls

Violin plots of the number of genes (nFeature_RNA, upper left), number of UMIs (nCount_RNA, upper right), mitochondria count percentage (percent.mt, lower left) and hemoglobin gene percentage (percent. HB, lower right) of all QC-passed cells in different tissues.

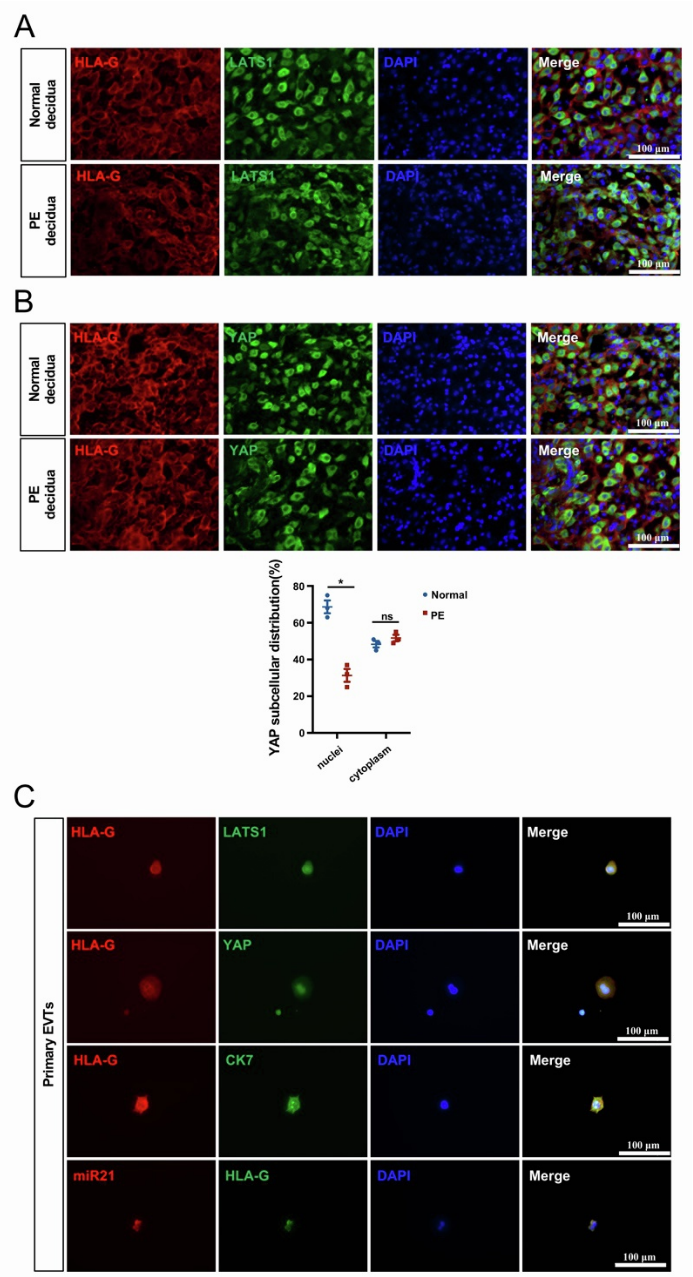

Figure S3. LATS1 and YAP expression patterns in human decidua and human primary extravillous trophoblasts (EVTs).

(A) IF staining of LATS1 (green) in human decidua collected from normal and PE-complicated pregnancies at term, EVTs were stained by HLA-G (red), nuclei were counterstained by DAPI (blue), scale bar 100 μm; (B) IF staining of YAP (green) and HLA-G (red) in human decidua, two-tailed t test, *$p<0.05$, scale bar 100 μm; (C) IF staining of LATS1 (green, upper), YAP (green, middle), CK7 (middle) and FISH of miR21 (red) in human primary EVTs isolated from first-trimester villi tissues, EVT marker was counterstained by HLG-G, scale bar 100 μm; data are presented as the mean ± SEM.

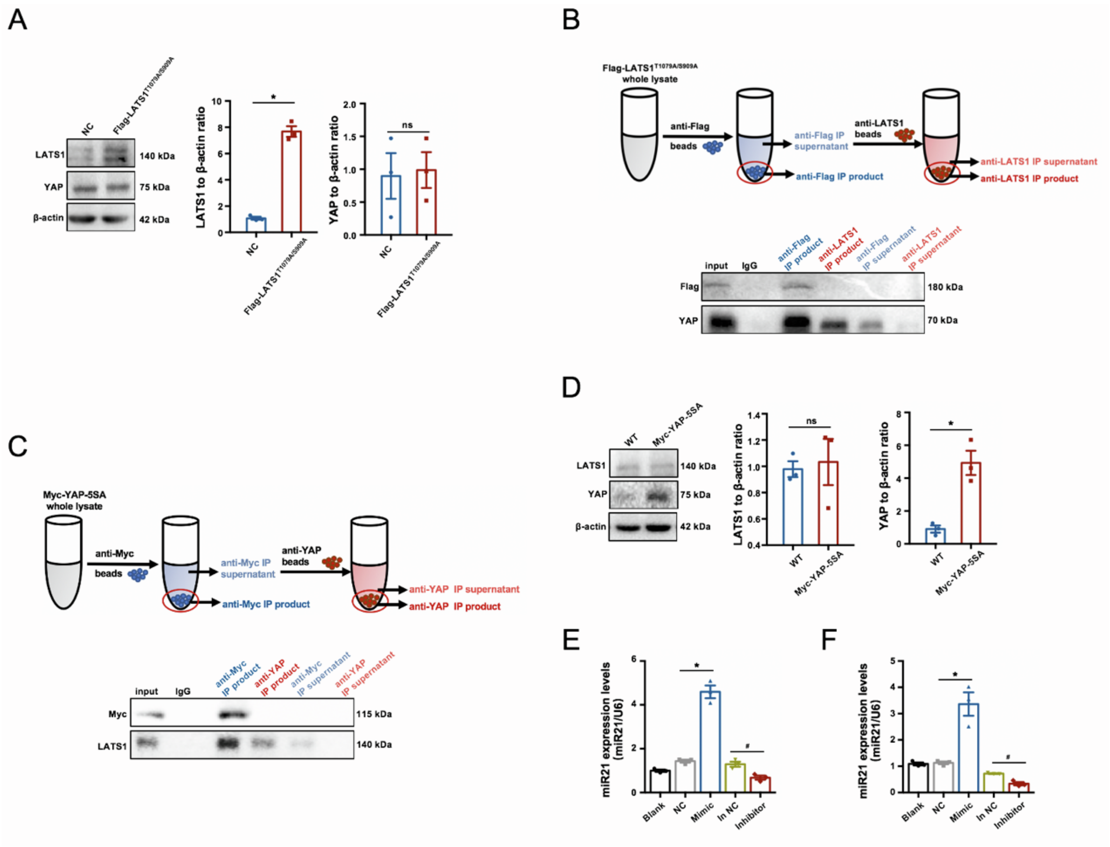

Figure S4. Establishment of Flag-LATS1$^{T1079A/S909A}$ and Myc-YAP-5SA HTR8/SVneo cells.

(A) Western blotting of LATS1 and YAP in Flag-LATS1$^{T1079A/S909A}$ NC (NC) and Flag-LAT1$^{T1079A/S909A}$ cells; n=3, two-tailed t test, *p<0.05; (B) The competitive binding of Flag-LATS1$^{T1079A/S909A}$ and WT LATS1 to YAP in Flag-LATS1$^{T1079A/S909A}$ cells assessed by successive Co-IPs; (C) The competitive binding of Myc-YAP-5SA and WT YAP to LATS1 in Myc-YAP-5SA cells assessed by successive Co-IPs; (D) Western blotting of LATS1 and YAP in the WT HTR8/SVneo cells and Myc-YAP-5SA cells; n=3, two-tailed t test, *p<0.05; Flag-LATS1$^{T1079A/S909A}$ cells and Myc-YAP-5SA cells were transfected with mimic NC (NC), inhibitor NC (in NC), miR21 mimic (mimic) or miR21 inhibitor (inhibitor) for 6 h, followed by culturing in fresh media for 48 h before any treatment or measurements. A blank control was included; RT-qPCR analysis of miR21 expression in (E) the Flag-LATS1$^{T1079A/S909A}$ cells and (F) the Myc-YAP-5SA cells; n=3 in each group, one-way ANOVA and Tukey's multiple comparison test, *p<0.05 vs. mimic NC; #p<0.05 vs. inhibitor NC; data are presented as the mean ± SEM.

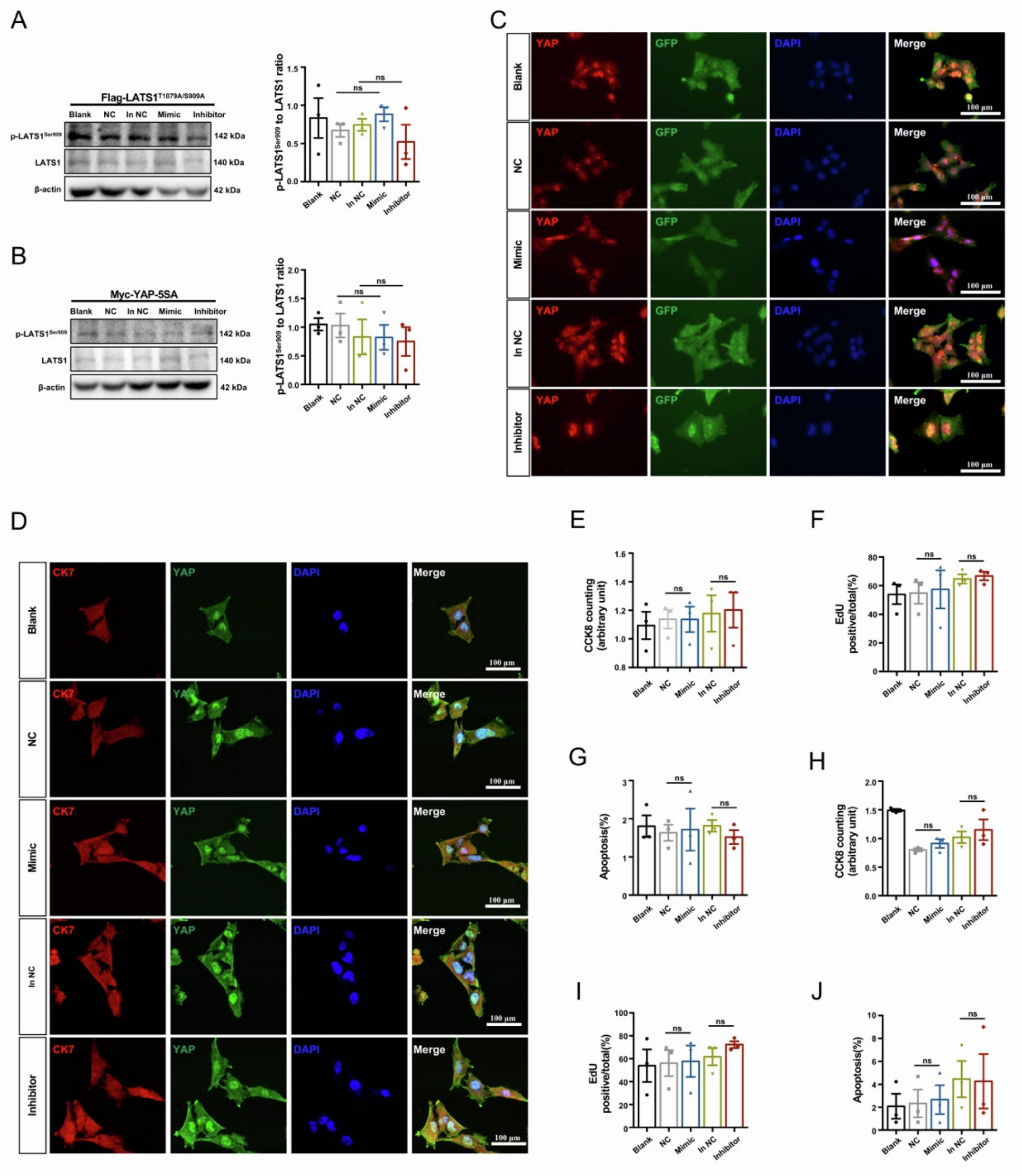

Figure S5. MiR21 activates YAP in phosphorylation-dependent ways.

Flag-LATS1$^{T1079A/S909A}$ cells and Myc-YAP-5SA cells were transfected with mimic NC (NC), inhibitor NC (in NC), miR21 mimic (mimic) or miR21 inhibitor (inhibitor) for 6 h, followed by culturing in fresh media for 48 h before any treatments or measurements. A blank control was included; Western blotting of p-LATS1^{Ser909} and LATS1 in (A) the Flag-LATS1$^{T1079A/S909A}$ cells and (B) Myc-YAP-5SA cells; (C) IF staining of YAP (red) and GFP (green) in the Flag-LATS1$^{T1079A/S909A}$ cells, scale bar 100 μm; (D) IF staining of YAP (green) and CK7 (red) in the Myc-YAP-5SA cells, scale bar 100 μm; (E) CCK-8 assay; (G) EdU assay; (G) flow cytometry for measuring apoptosis by staining Annexin V-FITC and PI in the Flag-LATS1$^{T1079A/S909A}$ cells; (H) CCK-8 staining, (I) EdU assay and (J) flow cytometry for measuring apoptosis by staining with Annexin V-FITC and PI of the Myc-YAP-5SA cells; n=3 in each group, one-way ANOVA and Tukey's multiple comparison test, *p<0.05 vs. mimic NC; #p<0.05 vs. inhibitor NC; data are presented as the mean ± SEM.

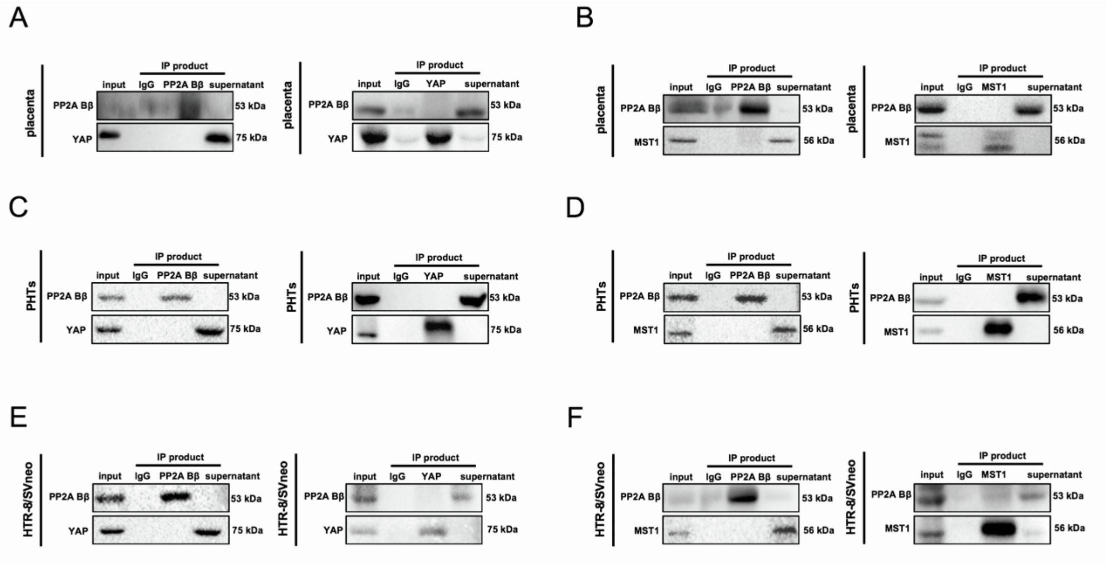

Figure S6. PP2A Bβ does not directly interact with MST1 and YAP.
Reciprocal Co-IP of (A) PP2A Bβ and YAP in human placenta; (B) PP2A Bβ and MST1 in human placenta; (C) PP2A Bβ and YAP in PHTs; (D) PP2A Bβ and MST1 in PHTs; (E) PP2A Bβ and YAP in HTR8-S/Vneo cells; (F) PP2A Bβ and MST1 in HTR8-S/Vneo cells.

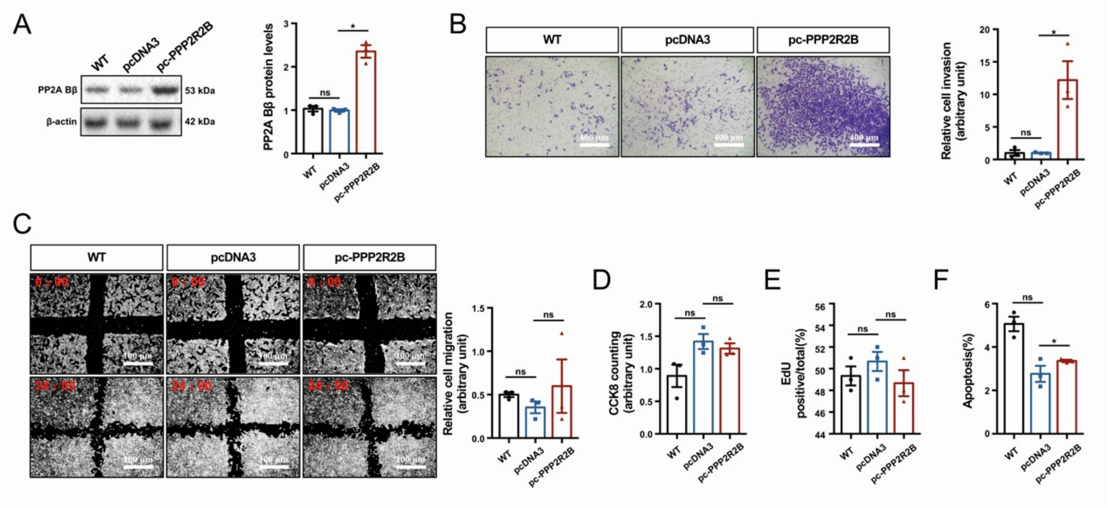

Figure S7. Establishment of PP2A Bβ-overexpressing HTR8/SVneo cells.
HTR8/SVneo cells were transfected with pcDNA3 or pc-*PPP2R2B* plasmids for 6 h, followed by culturing in fresh media for 48 h before any treatments and measurements. A WT control was included. (A) Western blotting of PP2A Bβ in the WT, *PPP2R2B* NC (pcDNA3) and *PPP2R2B*-overexpressing (pc-PPP2R2B) groups; n=3, one-way ANOVA, ns: nonsignificant, *$p<0.05$ vs. pcDNA3; representative images and statistics of (B) Matrigel Transwell assays, scale bar 400 μm, and (C) wound-healing assays, scale bar 100 μm; and (D) CCK-8 staining, (E) EdU assay and (F) flow cytometry for measuring apoptosis by staining with Annexin V-FITC and PI; n=3 in each group, one-way ANOVA and Tukey's multiple comparison test, ns: nonsignificant, *$p<0.05$ vs. pc-DNA3; data are presented as the mean ± SEM.

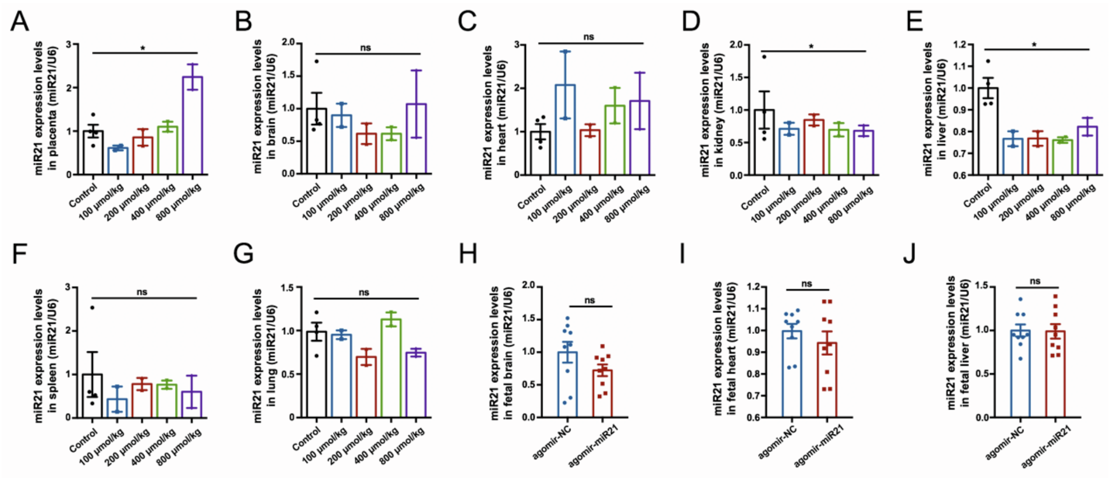

Figure S8. Dose gradient of agomir-miR21 nanoparticles specifically elevated miR21 in mouse placentae.

Different maternal organs were collected on E18.5 from dams treated with various dosages of agomir-miR21 nanoparticles; RT-qPCR of miR21 in (A) placenta; (B) brain; (C) heart; (D) kidney; (E) liver; (F) spleen; (G) lung; n=4 in the control group, n=2 in another agomir-miR21 group; one-way ANOVA test, *p<0.05 vs. the control group; Different fetal organs from fetuses were collected on E18.5 from dams in the agomir-NC or agomir-miR2 group; RT-qPCR of miR21 in (H) fetal brain; (I) fetal heart and (J) fetal liver; n=9 fetuses from 9 dams in each group; two-tailed t test, ns: nonsignificant; data are presented as the mean ± SEM.

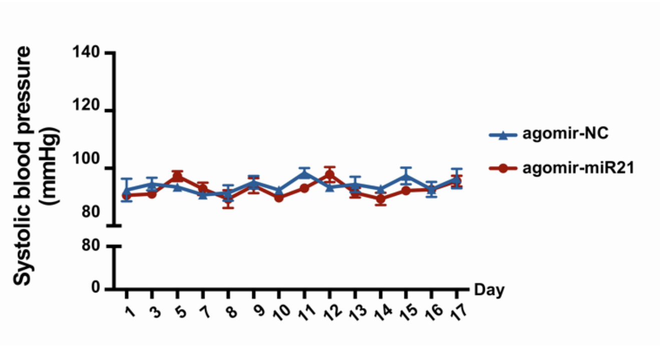

Figure S9. Blood pressure measurement of nonpregnant mice with agomir-miR21-nanoparticle treatment.

Systolic blood pressure of nonpregnant female mice treated with placenta-specific nanoparticles containing agomir-NC or agomir-miR21 from day 7 (D7) to day 9 (D9) through tail vein injection, n=3, two-way ANOVA and Tukey's multiple comparison test. Data are presented as the mean ± SEM.
